# Next-Generation Proteomics of Brain Extracellular Vesicles in Schizophrenia Provide New Clues on the Altered Molecular Connectome

**DOI:** 10.3390/biomedicines12010129

**Published:** 2024-01-08

**Authors:** Cristina Lorca, María Fernández-Rhodes, Jose Antonio Sánchez Milán, María Mulet, Félix Elortza, Alfredo Ramos-Miguel, Luis F. Callado, J. Javier Meana, Maria Mur, Iolanda Batalla, Elisabet Vilella, Aida Serra, Xavier Gallart-Palau

**Affiliations:** 1 Biomedical Research Institute of Lleida Dr. Pifarré Foundation (IRBLLEIDA), Neuroscience Area, +Pec Proteomics Research Group (+PPRG), University Hospital Arnau de Vilanova (HUAV), 80 Av. Rovira Roure, 25198 Lleida, Spain; cristinalorca92@gmail.com (C.L.); mfernandezr@ibecbarcelona.eu (M.F.-R.); jsanchez@irblleida.cat (J.A.S.M.); mmf37@alumnes.udl.cat (M.M.); 2 Department of Medical Basic Sciences, Biomedical Research Institute of Lleida Dr. Pifarré Foundation (IRBLLEIDA), +Pec Proteomics Research Group (+PPRG), University of Lleida (UdL), 25198 Lleida, Spain; 3 Proteomics Platform, CIC bioGUNE, Basque Research and Technology Alliance (BRTA), CIBERehd, Science and Technology Park of Bizkaia, 48160 Derio, Spain; felortza@cicbiogune.es; 4 Department of Pharmacology, University of the Basque Country UPV/EHU, 48940 Leioa, Spain; alfredo.ramos@ehu.eus (A.R.-M.); lf.callado@ehu.eus (L.F.C.); javier.meana@ehu.eus (J.J.M.); 5 Biocruces Bizkaia Health Research Institute, 48903 Barakaldo, Spain; 6 Centro de Investigación Biomédica en Red en Salud Mental CIBERSAM, Instituto de Salud Carlos III, 43206 Reus, Spain; 7 Psychiatry Department, Hospital Universitari Santa Maria, Medicine Department, Universitat de Lleida (UdL), 25198 Lleida, Spain; mmur@gss.cat (M.M.); ibatalla@gss.cat (I.B.); 8 Hospital Universitari Institut Pere Mata, Institut Investigació Sanitària Pere Virgili (IISPV)-CERCA, Universitat Rovira i Virgili, 43206 Reus, Spain; 9 Department of Psychology, University of Lleida (UdL), 25001 Lleida, Spain

**Keywords:** extracellular vesicles, molecular exchange, neuroinflammation, systems biology, immunoglobulins, brain antibodies, psychiatry, psychotic spectrum

## Abstract

Extracellular vesicles (EVs) are tiny membranous structures that mediate intercellular communication. The role(s) of these vesicles have been widely investigated in the context of neurological diseases; however, their potential implications in the neuropathology subjacent to human psychiatric disorders remain mostly unknown. Here, by using next-generation discovery-driven proteomics, we investigate the potential role(s) of brain EVs (bEVs) in schizophrenia (SZ) by analyzing these vesicles from the three post-mortem anatomical brain regions: the prefrontal cortex (PFC), hippocampus (HC), and caudate (CAU). The results obtained indicate that bEVs from SZ-affected brains contain region-specific proteins that are associated with abnormal GABAergic and glutamatergic transmission. Similarly, these vesicles from the analyzed regions were implicated in synaptic decay, abnormal brain immunity, neuron structural imbalances, and impaired cell homeostasis. Our findings also provide evidence, for the first time, that networks of molecular exchange (involving the PFC, HC, and CAU) are potentially active and mediated by EVs in non-diseased brains. Additionally, these bEV-mediated networks seem to have become partially reversed and largely disrupted in the brains of subjects affected by SZ. Taken as a whole, these results open the door to the uncovering of new biological markers and therapeutic targets, based on the compositions of bEVs, for the benefit of patients affected by SZ and related psychotic disorders.

## 1. Introduction

Schizophrenia (SZ) is a severe mental disorder that involves acute psychoticism and is estimated to affect 21 million people worldwide [1]. Individuals diagnosed with SZ commonly show impairment of the superior abilities of thinking, speech, emotional regulation, and social cognition [2]. The prevalence of the disorder shows a global rising tendency and involves a high disability rate for the affected individuals [1]. Thus, the personal and social burden of SZ is devastating in multiple facets, and nearly one-half of the subjects present refractory forms of the disease [3], which hardly respond to any available pharmacological treatments.

Multiple epidemiological culprits seem to be implicated in the origin of the disorder, including genetic, biologic, and environmental factors [3]. Early childhood trauma, the accumulation of traumatic events, and drug abuse are considered highly relevant factors in the erosion of the human capacity of resilience and in triggering first-episode psychosis (FEP), a stage of illness often preceding SZ [4]. Indeed, several large-scale genetic studies have already been performed to decipher the highly heritable rate of the disorder [5,6]. Multiple genetic variants, including common variants and rare mutations, have been associated with SZ. Common genetic variants, including those with weak or no statistically significant association, can explain more than 20% of the liability to the disorder [5]. Rare mutations in genes such as SETD1A, CUL1, XPO7, GRIA3, GRIN2A, and RB1CC1 have also been identified to substantially increase the risk of SZ, as previously reviewed [7], and large copy number variants (CNVs) with large effect sizes have also been implicated in the disorder [8]. Lastly, new findings about genetic loci linked to SZ susceptibility have shed light on the pathophysiology of the condition [9].

Similarly, neurochemical attempts to characterize the molecular basis of the disorder have also been conducted, and the alteration of the dopaminergic brain networks have been encountered [10,11]. SZ seems to also be associated with states of increased inflammation and oxidative stress, as well as with alterations of the innate immunity [12]. At a wider scope, patients with SZ present neuroanatomical changes, including a progressive loss of brain gray matter, which appears to be consistent with increased aberrant pruning and a consequent decreased number of cortical glutamatergic synapses [13]. Similarly, patients with SZ display white matter microstructural abnormalities across multiple brain regions, with a significant reduction in fractional anisotropy and widespread connectivity deficits, indicating that the disorder may result from an impaired brain connectome [14]. Although connectome alterations involving key neuroanatomical structures, such as the prefrontal cortex (PFC), hippocampus (HC), and caudate (CAU), have been largely identified in the brains of subjects with SZ [15], by using in vivo imaging approaches, any feasible molecular substrate for these connectome alterations remains elusive.

Extracellular vesicles (EVs) are tiny vesicles that act as intercellular carriers and actively participate in molecular exchange and communication within the central nervous system [16]. We, and other colleagues, have largely studied these vesicles using systems biology and have described their implications in multiple neurophysiological functions associated with aging-degenerative dementias and its therapeutic potential [17,18,19,20,21]. Proteome-wide analyses have shown that EV biogenesis is altered during preclinical AD, with the presence of specific proteins such as MHC class-type markers, prion protein (PrP), and amyloid protein (APP) [17]. Changes in the levels of AD-related proteins involved in vesicle endocytosis and the secretory pathways have been observed in both preclinical and symptomatic AD cases [17,18]. Furthermore, EVs from AD patients have been found to contain altered levels of cytokines, pS396 tau, Aβ1-42, and microRNA, which may reflect disease severity [22,23]. EVs have also been proposed as excellent platforms of nanovectors in neurologic diseases given their capacity to cross the blood–brain barrier [19,24,25]. It follows, hence, that these vesicles could play crucial functions in the altered brain connectome that is extensively found in SZ, while their role(s) in that disorder are still mostly unknown [26].

In order to shed light on the potential functions of EVs in the brain connectome of SZ, we perform here an in-depth characterization of the proteome compositions of post-mortem brain EVs (bEVs) from three different neuroanatomical regions that are broadly implicated in illness-related alterations in brain connectivity. The obtained data reflect the potential roles of these vesicles within the complexity of the psychiatric disorder and point towards the participation of bEVs in active brain networks of molecular exchange that become specifically altered in SZ.

## 2. Materials and Methods

### 2.1. Chemicals and Reagents

All chemicals and solvents were purchased from Sigma-Aldrich (St. Louis, MO, USA) unless otherwise specified. Water and acetonitrile (ACN) of liquid chromatography (HPLC) grade were purchased from Thermo Fisher Scientific (Thermo Fisher Chemical, Waltham, MA, USA). Sequencing-grade modified trypsin was purchased from Promega (Madison, WI, USA).

### 2.2. Human Brain Samples

All post-mortem brain specimens were collected during autopsies performed between 2010 and 2018 at the Basque Institute of Legal Medicine (Bilbao, Spain). Only samples with post-mortem delay < 24 h were included. Grey matter specimens from the dorsolateral PFC (DLPFC, approximating Brodmann area (BA) 9), hippocampus (HC), and caudate nucleus were carefully dissected, avoiding the white matter, and immediately stored at −80 °C until assay. Retrospective search into subjects’ medical records was conducted for antemortem diagnoses of SZ meeting DSM-IV or ICD-10 criteria. Clinical diagnoses of SZ were all performed by a board-certified psychiatrist of the Basque Healthcare System (Osakidetza). Cases with additional psychiatric or neurologic diagnoses, including a history of substance abuse, were excluded. Samples from 15 SZ cases meeting the above criteria were assayed in the present study. Each SZ case was paired to a matched (control, C) subject, matching case’s sex, age, and post-mortem delay, and with no evidence of psychiatric or neurological conditions, according to available antemortem medical records. Demographic characteristics of all C and SZ subjects are summarized in Table 1.

### 2.3. Preparation of Brain Tissues Prior to EVs Obtention

Brain tissues from each respective and previously detailed neuroanatomical region and subject were dissected, and any remaining meninges and large blood vessels were carefully inspected and removed. Dissected tissues were subsequently washed three times with 1× PBS for 30 min. Brain tissues from each neuroanatomical region and subject were randomly pooled to form three independent biological group replicates (*n* = 5; ~150 mg) per condition and neuroanatomical region, as we previously indicated [18,27]. Homogenization of dissected brain tissues was performed as previously described by our group [16] by using a Bullet Blender tissue homogenizer (Next Advance, Raymertown, NY, USA). Briefly, each sample was suspended in safe-lock tubes in a detergent-free homogenization buffer consisting of 100 mM ammonium acetate (AA) at pH 6.5 (500 µL), supplemented with protease inhibitor, and mixed with homogenization beads (150 mg, 0.9–2.00 mm magnetic particles) that were previously washed three times with 1× PBS for 30 min. Homogenization was then conducted in four cycles of 5 min each. At the end of each cycle, the homogenate was centrifuged at 15,000× *g* for 10 min, and the supernatants were collected. Afterwards, 300 μL of homogenization buffer was added, and the process was repeated. The intensity of the first two cycles was medium, and maximum intensity was used in the last two cycles. All of the procedures were performed at 4 °C.

### 2.4. Enrichment of Brain EVs by PROSPR

bEVs were enriched from the detergent-free brain homogenates by using PROSPR as we previously detailed [16]. Briefly, EV-containing homogenates were mixed with a fourfold volume of chilled acetone (−20 °C), vortexed and centrifuged at 5000× *g* for <1 min. Supernatants containing the hydrophobic EV fraction were then concentrated to near-dryness using a vacuum concentrator (Eppendorf AG, Hamburg, Germany) and stored at −80 °C until further use.

### 2.5. Characterization of Brain EVs using Nanoparticle Tracking Analysis

bEV fractions obtained from subjects with SZ and C subjects were subjected to in-depth characterization using a nanoparticle tracking analysis (NTA) as we previously described [18]. Briefly, bEV preparations were visualized and analyzed using a Nanosight NS300 with a sCMOS camera (Malvern Panalytical, Malvern, UK). Analysis parameters were set as follows: 60 s acquisition time, camera level 4, slider shutter of 50, slider gain of 100, FPS 32.5, syringe pump speed of 100, total volume per sample of 1 mL, viscosity of 0.906–0.910 cP, and temperature of ~24 °C. NTA was performed without establishing any restricted areas to the image fields by allowing for imaging and analysis of the full sample content.

### 2.6. Ultrastructural Characterization of Brain EVs

Representative bEVs fractions from SZ and C subjects were mounted on Cu-Formvar-carbon grids and kept for 20 min at room temperature (RT). Grids were then washed with HPLC water, and bEV preparations were fixed by using 1% glutaraldehyde in PBS for 5 min. The bEVs were subsequently stained with uranyl oxalate for 5 min, embedded in methyl-cellulose-uranyl-oxalate, and subsequently dried for permanent preservation. Electron micrographs were then imaged by using a Jeol Jem 1010 electron microscope at 80 kV. The obtained ultrastructural micrographs were then scale-calibrated, bi-leveled, and further analyzed by using the open software ImageJ (National Institutes of Health (NIH), Bethesda, MD, USA).

### 2.7. Processing of Brain EVs for Next-Generation Label-Free Proteomics

bEV samples were suspended in a lysis buffer composed of 16 M urea in 100 mM ammonium bicarbonate (ABB), subsequently incubated for 20 min at RT, and finally diluted 1:1 with HPLC water, as we previously indicated [17]. The bEV proteomes were then subjected to tryptic digestion, as previously described [19]. Briefly, bEV proteins were reduced using 10 mM dithiothreitol at 30 °C for 3 h, followed by alkylation using 20 mM iodoacetamide (IAA) for 1 h at room temperature in darkness. Next, urea was diluted to <1 M with 25 mM ABB, and the proteins were digested with trypsin overnight at 30 °C, with a 1:20 protein-to-enzyme ratio (*w*/*w*). Acidification with 0.5% final concentration of formic acid (FA) was used to quench the reaction. Waters Sep-Pak 50 mg C18 cartridges (Waters, Milford, MA, USA) were then used for to desalt the peptides, and peptide recovery was achieved by using a 70% ACN concentrated buffer. Finally, eluted peptides were concentrated by using a vacuum concentrator.

### 2.8. Four-Dimensional Proteomics of Brain EVs

Desalted bEVs and digested proteome samples were resuspended in 0.1% FA prior to LC-MS/MS analysis by using an EVOSEP liquid chromatographic instrument (EVOSEP, Odense, Denmark) at 300 nL·min^−1^. Samples were run by performing an 88 min gradient (15 samples per day). The EVOSEP liquid chromatographer was coupled online to the state-of-the-art timsTOF Pro mass spectrometer (Bruker Daltonics, Billerica, MA, USA), and the samples were analyzed by using four-dimensional (4D) parallel accumulation–serial fragmentation (PASEF) data acquisition, as previously detailed [19,28].

### 2.9. Bioinformatics and Data Analysis

Bioinformatic analysis of the obtained bEV 4D proteomics raw data was carried out, as previously indicated [29], by using the specialized proteomics suite software PEAKS Studio X (Bioinformatics Solutions INC., Waterloo, Canada). Precursor ion tolerance was set to 10 ppm, and fragment ion tolerance was set to 0.05 Da. Trypsin was set as proteolytic enzyme for database searching, and carbamidomethylation of Cys residues was set as fixed modification. The human Uniprot database (downloaded on 3 February 2023, containing 140,065 protein sequences) was used for the identification of proteins. Decoy fusion, FDR < 1%, was established for protein identification in all samples, and trypsin with cleavage on at least one end was set as a proteolytic enzyme. The bEV proteome data were exported to Microsoft Excel CSV files, and in-house-generated macros were created and used for further analysis.

Label-free relative quantification of bEV proteins between conditions and brain regions was performed based on spectral count, as previously reported [30,31,32,33], and the equality of variances was assessed using Levene’s test. The referred data were analyzed using parametric two-way ANOVA and by performing multiple comparisons and Fisher’s Least Significant Difference tests, with statistical significance set at *p* < 0.05 (95% confidence interval). Afterwards, additional Bonferroni correction for multiple comparisons was applied (*p* < 0.05). Proteins present in all three regions analyzed were subjected to correlation analysis, and only Pearson’s correlation coefficients ≥ ±0.8 were considered as indicators of strong interaction between the analyzed variables.

Additionally, Bioconductor “org.Hs.eg.db” package (version 3.16.0) and “clusterProfiler” package (version 4.6.2) were installed in R software (version 4.2.3) for Gene Ontology functional analysis and KEGG (Kyoto Encyclopedia of Genes and Genomes) pathway enrichment analysis.

### 2.10. Data Availability

All proteomics data generated for this study have been made publicly available through the specialized repository, PRIDE, with the following identifier: PXD042732. The free availability and re-usage of human brain generated systems biology data, such as these in this study, have been encouraged by the scientific community to openly contribute to the progress of the neuroscientific understanding of the human brain and its diseases [34].

## 3. Results

### 3.1. Morphometric Characteristics of bEVs in Schizophrenia

**Figure 1:**
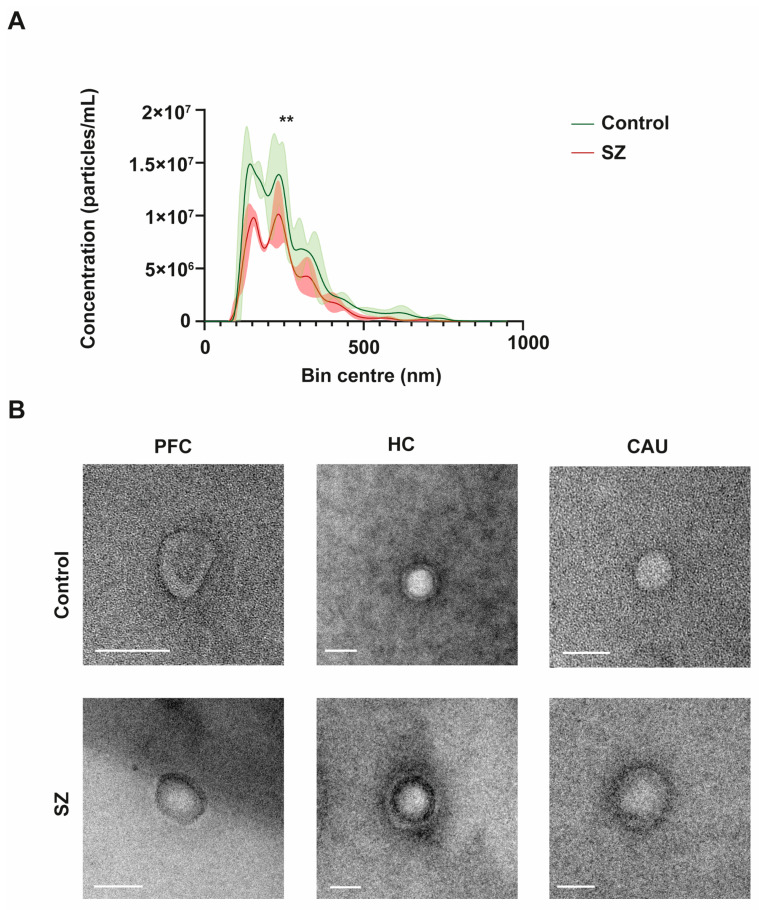
Morphological and ultrastructural characterization of brain extracellular vesicles (bEVs) obtained from post-mortem brain tissues. (**A**) Average size distribution profiles of bEVs obtained using nanoparticle tracking analysis (NTA) of bEVs from the prefrontal cortex (PFC) region of controls (C) and subjects with schizophrenia (SZ). Captures refer to the average distribution obtained from ten independent size/concentration distribution runs. Standard deviation of the mean is shaded in blue. (**B**) Representative micrographs of bEVs, obtained by transmission electron microscopy (TEM) from the PFC, hippocampus (HC), and caudate (CAU) regions of C subjects (upper micrographs) and of subjects with SZ (lower micrographs). Scale bar in Figure 1B represents 100 nm. ** indicates significant statistical differences (*p* < 0.0001).

To initially define the specific properties of bEVs, we performed an in-depth morphometric characterization of these vesicles in the brains of the controls (C) and of the subjects with SZ. The bEVs showed a mean concentration of 1.14 × 10^12^ particles/mL and an average diameter size of 255.73 nm (Figure 1A). Regarding the diameter size, there were no discernible variations between the groups under analysis; nevertheless, the mean concentration of bEVs showed a significant difference, with the C group’s bEVs exhibiting a greater concentration of particles (Figure 1A). Subsequently, we performed an ultrastructural analysis of bEVs using transmission electron microscopy (TEM) to define the predominant morphology and purity of the analyzed bEV preparations. As shown in the representative micrographs of Figure 1B, the EVs obtained showed predominant spherical morphology without any apparent differences observed between the C and subjects with SZ. Of note, the ultrastructural study also confirmed the absence of any appreciable particle contamination in the obtained bEV preparations (Figure 1B).

### 3.2. Molecular Compositions of bEVs in Schizophrenia

**Figure 2:**
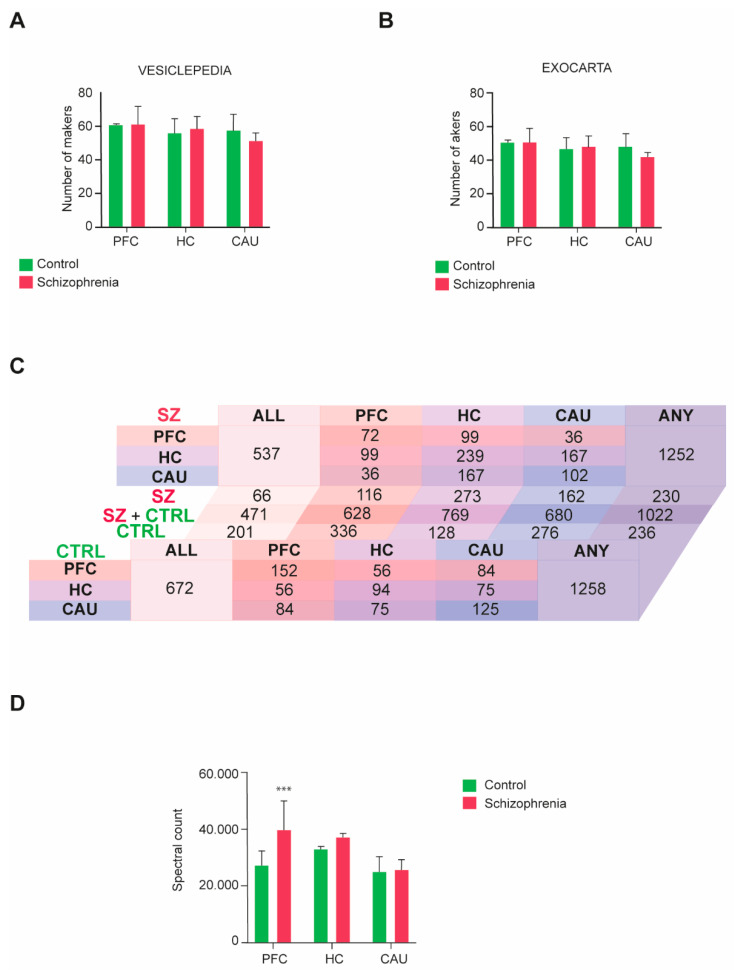
Molecular characterization performed by four-dimensional liquid chromatography proteomics of brain extracellular vesicles (bEVs) of the three analyzed brain regions, prefrontal cortex (PFC), hippocampus (HC), and caudate (CAU). (**A**,**B**) Presence of microvesicle markers in the proteomes of bEVs obtained from subjects with schizophrenia (SZ) and control (C) subjects. Parallel analysis of the obtained proteome data was performed in (**A**) with the top 100 protein markers curated in the specialized microvesicle data repository, Vesiclepedia, and in (**B**), it was performed with the specialized exosomal data repository, Exocarta. (**C**) Diagram table indicating the number of common and unique proteins present in the bEV proteomes of the analyzed brain regions, PFC, HC, and CAU, from C subjects and subjects with SZ. Salmon tones represent common proteins to the PFC region. Purple tones represent common proteins to the HC region, and blue tones represent common proteins to the CAU region. ALL indicates the number of proteins commonly present in the three evaluated regions, and ANY indicates the total number of proteins considering all identified proteins between the three evaluated regions. (**D**) Mean average cumulative proteome levels in bEVs of the three analyzed brain regions, PFC, HC, and CAU, from C subjects and subjects with SZ. *** indicates statistical significance at *p* < 0.001. Error bars represent standard deviation of the mean.

We then performed a four-dimensional unbiased discovery-driven characterization of the obtained bEV proteomes to define the molecular composition(s) of these vesicles in the three analyzed brain regions (PFC, HC, and CAU) from the C group and subjects with SZ (Supplementary Datasets S1 and S2). The obtained bEV proteomes data were subsequently matched to the data curated in the specialized EV database repositories, Exocarta and Vesiclepedia, as we previously indicated [16,17], to define the quality of the bEV preparations and identify any potential differences regarding the portion of exosomes and microvesicles present. Consistent identification between 70 and 90 percent of the top 100 exosomal and microvesicle markers, respectively curated in these databases, was achieved throughout the analyzed groups and brain regions (Figure 2A,B). Additionally, no significant differences were observed regarding the presence of EV markers between the analyzed groups and brain regions (Figure 2A,B).

The presence of common and unique specific proteins across groups and brain regions was also analyzed in the profiled bEV preparations (Figure 2C). No differences regarding the number of proteins present in bEV proteomes throughout the analyzed groups were detected, with a total of 1258 proteins being identified in the bEV proteomes of the C subjects and 1252 proteins being identified in the bEV proteomes of subjects with SZ (Figure 2C; Supplementary Datasets S1 and S2). However, whereas 672 proteins were identified as common to all brain regions in the bEV proteomes of the C subjects, only 537 were encountered in the bEV proteomes of the subjects with SZ (Figure 2C). Of note, 201 proteins of these that are common to all brain regions were unique to the bEVs of the C subjects, whilst 66 proteins were unique to the bEVs from the subjects with SZ (Figure 2C). Similarly, 57 proteins were uniquely identified in the PFC-bEVs, 206 proteins were uniquely identified in the HC-bEVs, and 86 proteins were uniquely identified in the CAU-bEVs of subjects with SZ (Figure 2C). Complete lists of these common and unique proteins identified in bEVs are, respectively, included in Supplementary Dataset S3 and Supplementary Tables S1–S5.

The total proteome bEVs levels were also analyzed in the three brain regions of the C group and subjects with SZ. A significant upregulation affecting the total proteome of bEVs in the PFC of subjects with SZ was observed (Figure 2D), whilst the bEV proteomes from the rest of brain regions scrutinized did not show significant alterations.

### 3.3. Schizophrenia-Linked Alteration of bEV Proteomes

**Figure 3:**
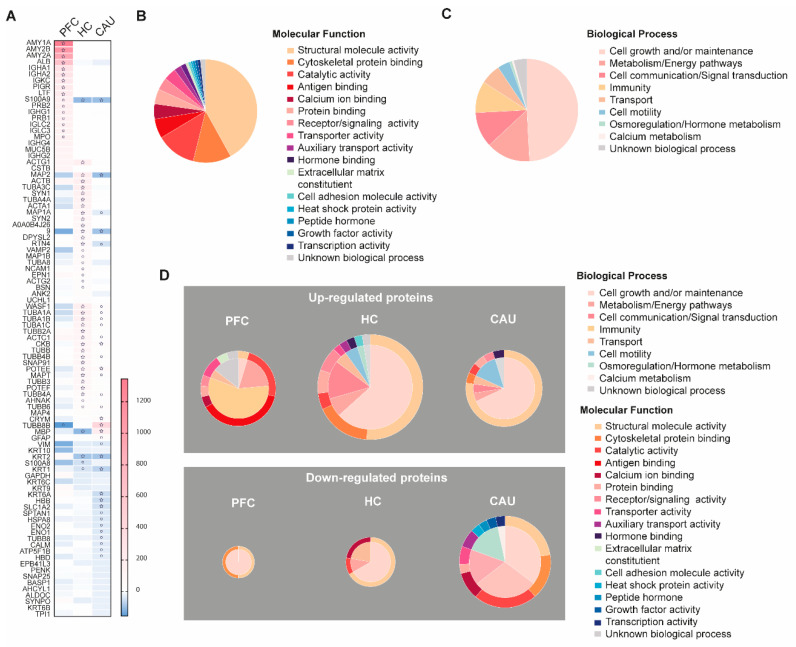
Depiction of the disorder-specific modulation, identified by four-dimensional proteomics, affecting the molecular compositions of brain extracellular vesicles (bEVs) in the three analyzed brain regions (prefrontal cortex (PFC), hippocampus (HC), and caudate (CAU)) of subjects with schizophrenia (SZ). (**A**) Heatmap showing significant modulation of bEV proteins from each, respectively, analyzed brain region of SZ brains. Proteins are indicated by gene symbol. (**B**) Sectorial graph showing the molecular functional classification of the proteins significantly modulated in bEVs of subjects with SZ. (**C**) Sectorial graph showing the biological process classification of the proteins significantly modulated in bEVs of subjects with SZ. (**D**) Sectorial graphs showing molecular functional classification (outer graph circle) and biological process classification (inner graph circle) of the proteins significantly modulated in bEVs of subjects with SZ obtained from the analyzed brain regions (PFC, HC, and CAU). Upper sectorial graphs depict significantly upregulated bEV proteins, whereas lower sectorial graphs depict significantly downregulated bEV proteins. ° symbol in the heatmap indicates significance *p* < 0.001; ☆ symbol in the heatmap indicates significance by Bonferroni corrected *p*-value (*p* < 0.05).

We then analyzed whether some of the bEV proteins in these subjects alter their levels throughout the three analyzed brain regions. The proteins that were identified with significantly altered regulation in these analyses are detailed in Figure 3A. These proteins, hereafter referred to as SZ-altered bEV proteins, were subsequently subjected to functional categorization considering their role(s) in specific molecular functions and biological processes, as shown in Figure 3B,C. We found that larger portions of these SZ-altered bEV proteins contribute to cell growth and homeostatic maintenance as biological processes and to the maintenance of cell structure as molecular function (Figure 3B). A large subset of these proteins was also associated with the malfunction of cell metabolism, brain immunity, and calcium homeostasis (Figure 3C).

Specifically, as shown in Figure 3A, the significant downregulation of structural proteins in bEVs from subjects with SZ, including actin (ACTA1), tubulins (TUBA1A, TUBA1B, TUBA1C, TUBB4A, TUBB6, and TUBB8B), cell adhesion (NCAM1), and microtubule-linked proteins (MAP1B, MAP4, MAP6, and MAPT), was observed. Of note, abnormal downregulation in the bEVS of subjects with SZ was also affecting essential structural and vesicular proteins associated with active synaptic densities and spines, including bassoon (BSN), synaptopodin (SYNPO), synapsins (SYN1 and SYN2), and SNAP 25 (Figure 3A). Conversely, the upregulation of immunoglobulins (IGHA1, IGHA2, IHKC, IGHG1, IGLC2, IGLC3, IGHG4, and IGHG2) was clearly identified in the disordered bEV proteomes of subjects with SZ (Figure 3A). Worthy of note, these identified bEV protein alterations from subjects with SZ were primordially observed in the PFC bEV proteomes and were not mirrored in the other brain regions that were analyzed (Figure 3A). Finally, it is worthy of mention that significant downregulations of the myelin protein (MBP) and the astroglial marker (GFAP) were also observed in the bEV proteomes of the subjects with SZ (Figure 3A).

A functional categorization of the significantly altered bEV proteomes of the subjects with SZ was also performed, as shown in Figure 3D. The upregulation of a subset of bEV proteins involved in immunity, antigen binding, and cellular transport was predominant in the PFC region of subjects with SZ (Figure 3D). Furthermore, the upregulation of a subset of bEV proteins involved in cell motility, cell maintenance, and cell structure was also predominant in the HC region of subjects with SZ (Figure 3D). Similarly, the upregulation of a high portion of bEV proteins involved in the maintenance of cell structure, cell growth and maintenance, cellular calcium homeostasis, cellular metabolism, and cellular transport was predominant in the CAU region of subjects with SZ (Figure 3D). On the contrary, the downregulation of a small subset of bEV proteins involved in cell maintenance and structure was observed in the PFC of subjects with SZ (Figure 3D). The downregulation of a substantial portion of bEVs proteins involved in cellular calcium homeostasis, catalytic functions, cell structure, cell maintenance, and transport was also predominant in the HC region of subjects with SZ (Figure 3D). Finally, a large subset of bEV proteins involved in catalytic functions, cellular structure, cell adhesion, growth factors, genetic transcription, hormonal metabolism, and cellular energy and metabolism was predominant in the CAU region of subjects with SZ (Figure 3D).

Functional categorization was also performed for the unique bEV region-exclusive proteins identified in the brains of subjects with SZ, as included in Table 2. Of note, we found several of these unique SZ-bEV proteins in the PFC involved in GABAergic synaptic transmission, while several of these in the HC are involved in glutamatergic transmission (Table 2). Additionally, several of these unique SZ-bEV proteins in the HC and CAU have been previously identified as being involved in neuronal impairment (Table 2).

### 3.4. Schizophrenia-Linked Alteration of the EV-Mediated Brain Connectome

**Figure 4:**
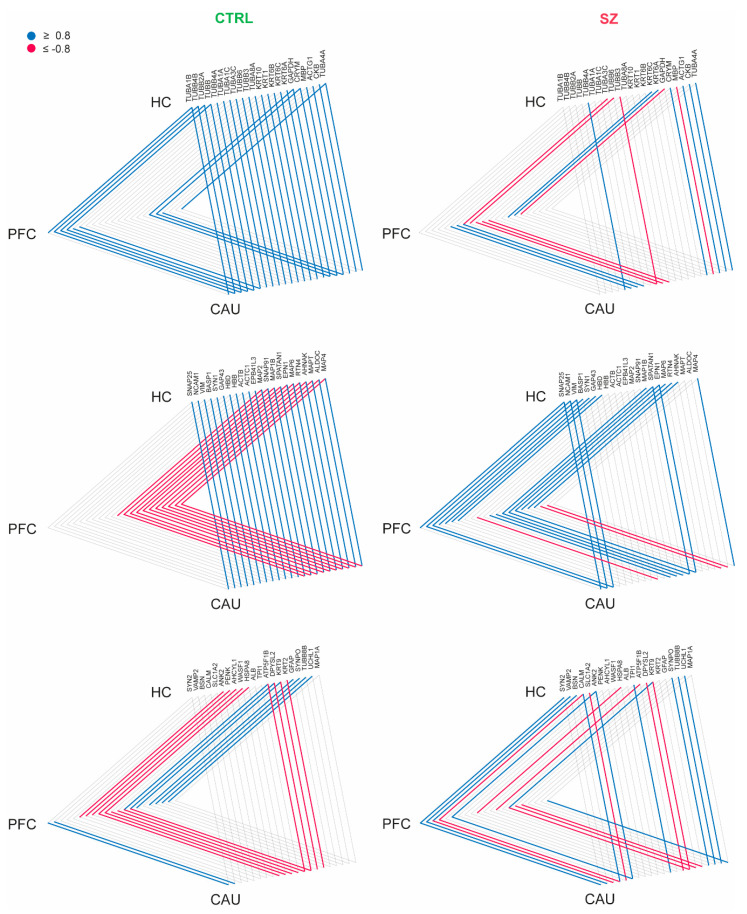
Interaction analysis of brain extracellular vesicle (bEV) proteins identified in the analyzed brain regions (prefrontal cortex (PFC), hippocampus (HC), and caudate (CAU)) of the controls (C) and subjects with schizophrenia (SZ). Proteins are represented collectively in three triangle diagrams per condition (C and SZ) for display purposes. Brain regions are represented at the triangle apexes. Lines in blue and red between apexes indicate strong positive correlation and strong negative correlation, respectively, for each specific protein in bEVs between brain regions (connected apexes). Only proteins significantly modulated (*p* < 0.05) in bEVs of subjects with SZ were included in the analysis, and only strong correlations (r ≥ ±0.8) are depicted.

To investigate whether bEVs exert any potential roles in the brain connectome linking the PFC, HC, and CAU regions in the brains of C subjects and subjects with SZ, we first focused on determining the proteins consistently identified in the three analyzed brain regions in our study; subsequently, we analyzed the presence of any strong correlation affecting the levels of these proteins in the analyzed brain regions. In these analyses, we found a strong interaction (r ≥ ±0.8) involving several cytoskeleton proteins (TUBA1B, TUBB2A, TUBB, TUBA4A, MAP2, MAP6, and MAPT), structural keratins (KRT6B and KRT6C), myelin basic protein (MBP), synapse-related proteins (SNAP25, SYN1, SNAP91, SYN2, VAMP2, and SYNPO), cell adhesion (NCAM1), glial (GFAP), and the neuronal collapsin (DPYSL2), among other proteins in the bEVs of the C subjects (Figure 4). Additionally, these data revealed that the CAU region seems to exert core effects on the molecular exchange mediated by bEVs between the three analyzed brain regions, with special emphasis on the predominant exchange with the HC (Figure 4).

We then analyzed whether these brain connectome molecular patterns, which are detailed and mediated by bEVs in the non-psychotic brain, suffer any alteration in brains affected by SZ. Strikingly, we found that the referred connectome patterns involving bEV proteins in the PFC, HC, and CAU regions were lost in most of the protein cases (Figure 4), and in the case of MBP, DPYSL2 and the Tau protein, MAPT, were completely reversed, as can be observed in Figure 4. Furthermore, the data presented in Figure 4 are included in their raw form in Supplementary Dataset S4. Additionally, the core molecular exchanging effect attributable to the CAU region in the non-diseased brains, and in the brains affected by SZ, was almost unappreciable (Figure 4).

**Figure 5:**
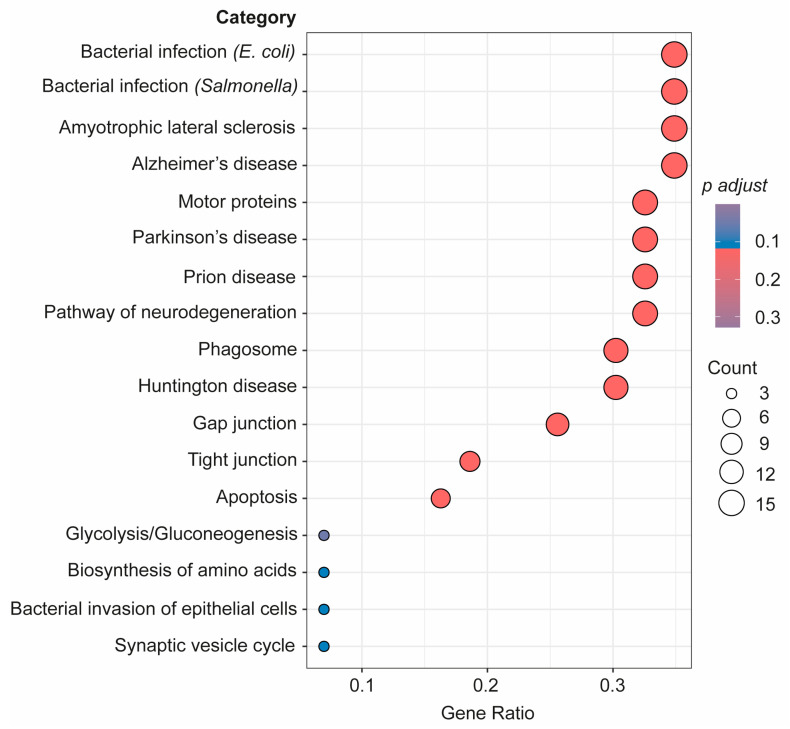
Advanced pathway analysis of the connectome proteins in bEVs identified as changed in SZ brains considering the three analyzed regions. Proteins were subjected to advanced pathway bioinformatic analysis in R through clusterprofiler in enrichGO package.

As seen in Figure 5, a further pathway analysis of the connectome proteins identified as changed in the bEVs of SZ brains was also conducted. According to our investigations, these proteins in the bEVs are involved in pathways that become dysregulated in various neurological illnesses, immunological responses to bacterial infections, in cellular death by apoptosis, glycolysis, and during synaptic vesicle cycle regulation (Figure 5). R bioinformatics was also used to categorize these proteins according to their predominant primary cellular origins (Table 3). According to this inquiry, neuronal origin accounted for the majority of the bEV proteins that were shown to likely be altered in the SZ bEV-mediated connectome (Table 3).

## 4. Discussion

This study timely addresses an existing gap in the research concerning bEVs in SZ, providing relevant findings. These findings not only contribute to a better understanding of the role(s) of bEVs in the neuropathology of SZ but also, to the best of our knowledge, directly indicate the implication of these vesicles in the connectome of healthy and diseased brains for the first time, which is an aspect that will be discussed in detail later.

As recently emphasized [26], it is imperative to improve our understanding of the potential role(s) of EVs in the pathophysiology of psychiatric disorders [35], specifically SZ, through the implementation of studies focused on the high-throughput proteomics characterization of these brain vesicles [26]. Following the foundation established by Efrat Levy and colleagues in 2012 [33,34], we pioneered the enrichment of EVs from brain tissues for their analysis by systems biology in 2016 [36,37]. These high-throughput technologies have been widely applied, both by us [16,17,18,38] and by other colleagues [39,40,41,42], to unravel the molecular basis of aging-associated neurodegeneration. However, its transition to SZ, which was still pending [26], is presented in this study.

Here, we did not find any specific morphological characteristics of bEVs that could be linked to SZ. A mean diameter size of ~250 nm, which is highly consistent with previous reports on bEVs [16,17,18,42], was predominantly encountered in C and SZ brains. Similarly, an ultrastructural analysis of the obtained bEV preparations by TEM revealed that these vesicles show spherical shapes that, in some cases, display the presence of a double membrane, features that are widely consistent with previous findings [36], and a characteristic trait of EVs that possess high circulatory capacity in biological fluids [19,35]. An in-depth analysis of the molecular markers defining bEVs also accounted for the quality and purity of the obtained bEV preparations, since between 70 and 90 percent of specific exosomal and microvesicle markers in our data successfully matched the top 100 markers in the EV-specialized database repositories, Exocarta [43] and Vesiclepedia [44]. Similarly, no clear differences were observed regarding the total number of proteins found in the bEV proteomes of the C subjects and subjects with SZ, but only in the number of unique bEV proteins associated with the regions analyzed in the brains of subjects with SZ, which included substantially less proteins compared to the non-diseased subjects. This fact could also be linked to the significantly lower concentrations of bEVs observed in SZ brains compared to C brains, which is a matter that requires further research. In line with this, the proteome levels of bEVs in the brains of subjects with SZ were significantly higher compared to those of the C subjects only in the PFC region. Hence, these findings indicate that the richness in protein variety that typically conforms bEVs in cognitively healthy conditions subsides in the brains of subjects affected by SZ; however, these vesicles in the PFC contain significantly higher cumulative proteome levels compared to those from non-diseased brains.

Following one of the main findings of this study, we focus our attention on the alteration of the levels of crucial synaptic proteins in the bEVs of SZ brains. Protein abundances were found to be altered in the bEVs of primordial cognitive cortical regions, such as PFC and HC. One of these proteins is the synaptic vesicle-associated protein bassoon, which was found to be specifically downregulated in the SZ bEVs of the PFC and upregulated in the SZ bEVs of the HC. Basson, as recently detailed by Montenegro-Venegas et al., organizes and regulates the active synaptic pools within glutamatergic synapses [45]. Similarly, synaptopodin, a protein that was downregulated in the SZ bEVs of the the PFC and CAU regions in our study, is crucial to provide long-term stability to active spines [46]. The SNARE (soluble N-ethylmaleimide sensitive factor attachment protein receptor) proteins, Synapsin 1 and SNAP-25, which were downregulated in the SZ bEVs of the PFC here, are also essential to sustain normal exocytosis at the presynaptic terminals. Of note, the MAP1B protein was also found to be downregulated in the SZ bEVs of the PFC region and upregulated in the SZ bEVs of the HC in our study. MAP1B, in neuronal EVs, is released following neuronal depolarization, and its presence may be implicated in the neurite removal and pruning of inactive synapses [47]. These findings, related to synaptic proteins in bEVs, indicate that these vesicles are potentially implicated in a broad picture of synaptic decay by directly affecting the PFC in the brains of subjects affected by SZ. The accumulation of dysfunctional synapses has been directly associated with cognitive decay in human dementias [48,49], and our findings suggest that bEVs would promote the proper maintenance of abnormal synapses only in the HC and not in the PFC. However, such an important finding ought to require further research. In line with this, an analysis of proteins unique to each respective brain region and to the SZ bEVs, and thus not present in the C bEVs nor in the bEVs of other regions analyzed in the brains of subjects affected by SZ, pointed to an exclusive presence of proteins related to GABAergic synapses in the PFC and to glutamatergic synapses in the HC of disordered brains. Whether these proteins might be part of a synergistic predominance linked to the psychiatric disorder affecting these specific brain regions, mapped through bEV fingerprinting, remains unknown. These findings may also contribute to explain the dichotomy that has been found in the brains of subjects with SZ regarding the hyperexcitability of the HC region [50] and the hypoexcitability of the PFC region [51], which is a fact that we also consider to be extremely worth further exploring.

Our data also indicate an exacerbated upregulation of antibody immunoglobulins in the bEVs of the PFC region in SZ brains. These molecules are typically produced by B cells and kept away from the central nervous system (CNS) in normal neurologic conditions [52]. Moreover, its presence within the CNS has been associated with the apparition of psychotic symptomatology secondary to autoimmune diseases [53,54]. Although the presence of auto-antibodies in the brains of subjects affected by SZ has been previously reported, the mechanisms involved in specific blood–brain barrier permeability for these molecules remains unknown [55]. The fact that bEVs become a source that explains the arrival mechanism and increase in immunoglobulins in the PFC of brains affected by SZ, based on the findings here, is highly compelling, and after further validation, it holds promise to contribute to identifying new therapeutic targets for the disorder.

Although bEVs seem to undoubtedly implicate in brain interregional molecular exchange, as recently indicated [35], clear evidence of that outcome in humans is still lacking. Of note, the findings reported in this study strongly support this relevant fact, for the first time, both in healthy and diseased brains. This study has shown that there is interaction affecting the levels of certain proteins that are commonly identified in bEVs in the three analyzed brain regions. This finding was interpreted as the uncovering of a potential active network of molecular exchange mediated by bEVs in the analyzed brains. Furthermore, our data show that bEVs in the CAU region show active exchange potentiality with HC and PFC in the cognitively normal brain, which is a significant discovery that needs more verification because it may have significant effects on how the brain communicates between its cells and domains under normal circumstances. Strikingly, we also observed that the potential active networks of molecular exchange between the three analyzed regions become altered in the analyzed brains from subjects with SZ, thus indicating that these active networks mediated by bEVs become disrupted in the disorder. Specifically, the myelin protein, MBP; the collapsin, DPYSL2; and the Tau protein, MAPT, were the relevant proteins clearly identified to be involved in that disrupted brain network mediated by EVs. All of these referred proteins were thus showing reversed strong correlations with the bEVs of subjects with SZ compared to those of C subjects.

## 5. Conclusions

Collectively, the findings reported in this study identify crucial proteins linked to the bEVs in SZ and reinforce the initial hypothesis about the implication of these vesicles in the neuropathology of the disorder. Additionally, our research identifies specific protein markers in brain EVs with the ability to circulate in biological fluids. These markers may prove to be useful in stratifying clinical patients and correlating the symptomatology of their disorders with the predominance of neuropathology in specific brain regions. The obtained results also contribute to the evidence suggesting that bEVs orchestrate molecular exchange in the human brain, introducing the novelty that alterations to this process occur in psychiatric diseases, particularly SZ. As such, these vesicles might also constitute the brain’s blueprint for the connectome changes previously discovered by neuroimaging studies. Lastly, they offer a fresh and extremely valuable source of potential therapeutic targets for the illness, which, altogether, merits further research.

## Figures and Tables

**Table 1:** Details of the human post-mortem brains analyzed in this study. ^a^ PMD refers to post-mortem delay expressed in hours.

Subject	Gender	Age at Death (y.o.)	PMD ^a^ (h)	Clinical Group
1	M	58	6	SZ
2	M	57	3	C
3	M	51	18	SZ
4	M	50	2	C
5	M	58	24	SZ
6	M	58	20	C
7	M	58	16	SZ
8	M	56	15	C
9	M	60	17	SZ
10	M	60	14	C
11	F	46	12	SZ
12	F	48	21	C
13	M	43	6	SZ
14	M	44	21	C
15	F	50	14	SZ
16	F	49	18	C
17	M	63	20	SZ
18	M	63	20	C
19	M	48	13	SZ
20	M	47	20	C
21	M	60	7	SZ
22	M	60	19	C
23	M	52	24	SZ
24	M	52	23	C
25	M	52	11	SZ
26	M	51	16	C
27	F	52	10	SZ
28	F	51	10	C
29	M	41	17	SZ
30	M	41	15	C

**Table 2:** Principal molecular functions of the proteins exclusively detected in the bEVs of each analyzed brain region from subjects with SZ. ^a^ Proteins are listed by gene symbol.

Region	Molecular Function	Protein List ^a^
**PFC-SZ**	Clathrin-coated vesicle	ATP6V1A, CD9, AP2B1, AP1B1
	Lysosomal membrane	ATP6V1A, AP2B1, LRP1, GNAI1, GNAI3, AP1B1
	Focal adhesion	GDI2, ARF1, CD9, TLN2, CAPN1, LRP1, PDPK1, RPS19, RPL4, CLASP1, MSN, TPM4
	Cell-substrate junction	GDI2, ARF1, CD9, TLN2, CAPN1, LRP1, PDPK1, RPS19, RPL4, CLASP1, MSN, TPM4
	Postsynaptic density	ARF1, PDPK1, RPS19, PDLIM5
	Gabaergic synapse	SLC12A5, GNAI3, GLUL, GNAI1
**HC-SZ**	Regulation of synaptic plasticity	SQSTM1, APOE, BRAF, SHISA6, RAPGEF2, ERC1, GRIA1, SHISA7
	Regulation of actin cytoskeleton organization	PFN2, GPM6B, SYNPO2, WASF3, BRAF, LIMCH1, CAPZB, RASA1, WDR1, WASH6P, RAC3, LMOD2
	Regulation of supramolecular fiber organization	PFN2, SYNPO2, APOE, WASF3, BRAF, LIMCH1, CAPZB, RPS3, RASA1, WDR1, WASH6P, LMOD2
	Purine ribonucleotide metabolic process	ADCY9, HINT1, NDUFAB1, HDAC4, VCP, OPA1, ATP5ME, DLG2, NDUFV3, SDHD, ATP1B1, ADSS2
	Regulation of actin filament-based process	SRI, PFN2, GPM6B, SYNPO2, WASF3, BRAF, LIMCH1, CAPZB, RASA1, WDR1, WASH6P, RAC3, LMOD2
	Synapse organization	GAP43, PFN2, APOE, CNKSR2, WASF3, SHISA6, VCP, ERC1, ELFN1, SHISA7, SNTA1, L1CAM, CLSTN1
	Regulation of trans-synaptic signaling	PFN2, SQSTM1, APOE, BRAF, SHISA6, RAPGEF2, ERC1, GRIA1, SHISA7, DLGAP2, DLGAP4, CACNA1A, PSMC5, CPLX3, CLSTN1
	Actin filament organization	PFN2, SYNPO2, MARCKSL1, WASF3, BRAF, LIMCH1, SHROOM2, SHROOM3, CAPZB, RASA1, ACTA1, WDR1, WASH6P, RAC3, MYO5A, SAMD14, LMOD2
	Glutamatergic synapse	GRIA1, ADCY9, GNAQ, CACNA1A, PRKCA, GLS
	Pathways of neurodegeneration—multiple diseases	GRIA1, VCP, BRAF, PRKCA, SDHD, KLC1, PSMC5, ACTR1B, PSMD4, GNAQ, NDUFAB1, NDUFV3, SQSTM1
**CAU-SZ**	Cell–cell adhesion via plasma membrane adhesion molecules	PCDHGB4, PCDHGB5, PCDHGB3, PCDHGA12, PCDHGB6, PCDHGA5, PCDHGA7, PCDHGA2, PCDHGB1, PCDHGA1, PCDHGC3, PCDHGB2, PCDHGA6, PCDHGA11, PCDHGA3, PCDHGB7, PCDHGA4, PCDHGC4, PCDHGA9, PCDHGA10, PCDHGA8
	Presynaptic active zone	STX1A, CANX, GPM6A, SLC32A1
	Synaptic vesicle	PENK, STX1A, SLC30A3, SEPTIN2, SLC32A1
	Dendritic spine	STRN, CALB1, STRN4, PPP1R9A, CANX, GPM6A, NOS1
	Neuron spine	STRN, CALB1, STRN4, PPP1R9A, CANX, GPM6A, NOS1
	Mitochondrial protein-containing complex	HSPA9, TIMM13, ATP5F1C, TIMM8A, NDUFS5, NDUFA8, DLAT
	Mitochondrial inner membrane	HSPA9, TIMM13, ATP5F1C, TIMM8A, NDUFS5, NDUFA8, CKMT2, SFXN5, LETM1
	Calmodulin binding	STRN, STRN4, MYO1C, SNTB2, PCNT, NOS1, CNN1
	Actin binding	PHACTR1, GMFB, PPP1R9A, MYO1C, CTNNA1, CTNNA2, SNTB2, CNN1
	Pathways of neurodegeneration—multiple diseases	NDUFA8, TUBAL3, NDUFS5, VDAC3, NOS1, ATP5F1C, STX1A

**Table 3:** Functional categorization of potential altered connectome proteins in bEVs of SZ brains based on the cellular origin categorized using bioinformatics (Clusterprofiler function of enrichGO package in R).

ID	Symbol	Gene Name	Origin
P60709	ACTB	actin beta	Neuronal/Glial
B3KPP5	ACTC1	actin alpha cardiac muscle 1	Neuronal
P63261	ACTG1	actin gamma 1	Neuronal
Q01484	ANK2	ankyrin 2	Neuronal
P80723	BASP1	brain abundant membrane attached signal protein 1	Neuronal
Q9UPA5	BSN	bassoon presynaptic cytomatrix protein	Neuronal
Q9Y2J2	EPB41L3	erythrocyte membrane protein band 4.1 like 3	Neuronal
Q9Y6I3	EPN1	epsin 1	Neuronal
P17677	GAP43	growth associated protein 43	Glial
P14136	GFAP	glial fibrillary acidic protein	Glial
P78559	MAP1A	microtubule-associated protein 1A	Neuronal
A2BDK6	MAP1B	microtubule-associated protein 1B	Neuronal
P11137	MAP2	microtubule-associated protein 2	Neuronal
P27816	MAP4	microtubule-associated protein 4	Neuronal
Q96JE9	MAP6	microtubule-associated protein 6	Neuronal
P10636	MAPT	microtubule-associated protein tau	Neuronal
P02686	MBP	myelin basic protein	Neuronal
P13591	NCAM1	neural cell adhesion molecule 1	Glial
A0A024R7V4	PENK	proenkephalin	Neuronal
Q9NQC3	RTN4	reticulon 4	Neuronal
A2A2U1	SLC1A2	solute carrier family 1 member 2	Glial
P60880	SNAP25	synaptosome-associated protein 25	Neuronal
O60641	SNAP91	synaptosome-associated protein 91	Neuronal
P17600	SYN1	synapsin I	Neuronal
A0A087X2E3	SYN2	synapsin II	Neuronal
Q8N3V7	SYNPO	synaptopodin	Neuronal
Q71U36	TUBA1A	tubulin alpha 1a	Neuronal
P68363	TUBA1B	tubulin alpha 1b	Neuronal
B7Z1K5	TUBA1C	tubulin alpha 1c	Neuronal
Q13748	TUBA3C	tubulin alpha 3c	Neuronal
P68366	TUBA4A	tubulin alpha 4a	Neuronal
B4DY90	TUBB	tubulin beta class I	Neuronal
Q13885	TUBB2A	tubulin beta 2A class IIa	Neuronal
Q13509	TUBB3	tubulin beta 3 class III	Neuronal
P04350	TUBB4A	tubulin beta 4A class IVa	Neuronal
P68371	TUBB4B	tubulin beta 4B class IVb	Neuronal
Q9BUF5	TUBB6	tubulin beta 6 class V	Neuronal
NA	TUBB8B	tubulin beta 8B	Neuronal
P09936	UCHL1	ubiquitin C-terminal hydrolase L1	Neuronal
F8WCA0	VAMP2	vesicle-associated membrane protein 2	Neuronal
P08670	VIM	vimentin	Glial

## Data Availability

All proteomics data generated for this study have been made publicly available through the specialized repository, PRIDE, with the following identifier: PXD042732.

## Supplementary Materials

The following supporting information can be downloaded at https://www.mdpi.com/article/10.3390/biomedicines12010129/s1, Supplementary Dataset S1: List of identified proteins in bEVs of C brains in each brain region. Supplementary Dataset S2: List of identified proteins in bEVs of SZ samples in each brain region. Supplementary Dataset S3: List of identified proteins in bEVs of control and SZ samples common to all brain regions; Supplementary Dataset S4: Data of the connectome analysis shown in Figure 4. On the right, average and standard deviation (SD) for every significative protein of bEVs consistently identified in the three analyzed anatomical brain regions. On the left, correlation matrix. Table S1: Proteins exclusively identified in brain extracellular vesicles (bEVs) of subjects affected by schizophrenia (SZ) in all analyzed brain regions. Table S2: Proteins exclusively identified in brain extracellular vesicles (bEVs) of control (C) subjects in all analyzed brain regions. Table S3: Proteins exclusively identified in bEVs of the PFC region in SZ. Table S4: Proteins exclusively identified in bEVs of the HC in SZ. Table S5: Proteins exclusively identified in bEVs of the CAU in SZ.

## References

[1] Charlson F.J., Ferrari A.J., Santomauro D.F., Diminic S., Stockings E., Scott J.G., McGrath J.J., Whiteford H.A. (2018). Global Epidemiology and Burden of Schizophrenia: Findings from the Global Burden of Disease Study 2016. Schizophr. Bull..

[2] Saleem A., Qurat Ul A., Akhtar M.F. (2022). Alternative Therapy of Psychosis: Potential Phytochemicals and Drug Targets in the Management of Schizophrenia. Front. Pharmacol..

[3] Nucifora F.C., Woznica E., Lee B.J., Cascella N., Sawa A. (2019). Treatment resistant schizophrenia: Clinical, biological, and therapeutic perspectives. Neurobiol. Dis..

[4] Huang Z.H., Hou C.L., Huang Y.H., He X.Y., Wang Q.W., Chen X., Wang Z.L., Wang S.B., Jia F.J. (2019). Individuals at high risk for psychosis experience more childhood trauma, life events and social support deficit in comparison to healthy controls. Psychiatry Res..

[5] Nakamura T., Takata A. (2023). The molecular pathology of schizophrenia: An overview of existing knowledge and new directions for future research. Mol. Psychiatry.

[6] Owen M.J., Sawa A., Mortensen P.B. (2016). Schizophrenia. Lancet.

[7] Golov A.K., Kondratyev N.V., Kostyuk G.P., Golimbet A.V.E. (2020). Novel Approaches for Identifying the Molecular Background of Schizophrenia. Cells.

[8] Zamanpoor M. (2020). Schizophrenia in a genomic era: A review from the pathogenesis, genetic and environmental etiology to diagnosis and treatment insights. Psychiatr. Genet..

[9] Liu C., Kanazawa T., Tian Y., Mohamed Saini S., Mancuso S., Mostaid M.S., Takahashi A., Zhang D., Zhang F., Yu H. (2019). The schizophrenia genetics knowledgebase: A comprehensive update of findings from candidate gene studies. Transl. Psychiatry.

[10] Iasevoli F., Avagliano C., D’Ambrosio L., Barone A., Ciccarelli M., De Simone G., Mazza B., Vellucci L., de Bartolomeis A. (2023). Dopamine Dynamics and Neurobiology of Non-Response to Antipsychotics, Relevance for Treatment Resistant Schizophrenia: A Systematic Review and Critical Appraisal. Biomedicines.

[11] Kesby J.P., Eyles D.W., McGrath J.J., Scott J.G. (2018). Dopamine, psychosis and schizophrenia: The widening gap between basic and clinical neuroscience. Transl. Psychiatry.

[12] Buckley P.F. (2019). Neuroinflammation and Schizophrenia. Curr. Psychiatry Rep..

[13] Calvin O.L., Redish A.D. (2021). Global disruption in excitation-inhibition balance can cause localized network dysfunction and Schizophrenia-like context-integration deficits. PLoS Comput. Biol..

[14] Kelly S., Jahanshad N., Zalesky A., Kochunov P., Agartz I., Alloza C., Andreassen O.A., Arango C., Banaj N., Bouix S. (2018). Widespread white matter microstructural differences in schizophrenia across 4322 individuals: Results from the ENIGMA Schizophrenia DTI Working Group. Mol. Psychiatry.

[15] Satterthwaite T.D., Vandekar S.N., Wolf D.H., Bassett D.S., Ruparel K., Shehzad Z., Craddock R.C., Shinohara R.T., Moore T.M., Gennatas E.D. (2015). Connectome-wide network analysis of youth with Psychosis-Spectrum symptoms. Mol. Psychiatry.

[16] Gallart-Palau X., Serra A., Sze S.K. (2016). Enrichment of extracellular vesicles from tissues of the central nervous system by PROSPR. Mol. Neurodegener..

[17] Gallart-Palau X., Guo X., Serra A., Sze S.K. (2020). Alzheimer’s disease progression characterized by alterations in the molecular profiles and biogenesis of brain extracellular vesicles. Alzheimers Res. Ther..

[18] Gallart-Palau X., Serra A., Hase Y., Tan C.F., Chen C.P., Kalaria R.N., Sze S.K. (2019). Brain-derived and circulating vesicle profiles indicate neurovascular unit dysfunction in early Alzheimer’s disease. Brain Pathol..

[19] Lorca C., Laparra M., Céspedes M.V., Casaní L., Florit S., Jové M., Mota-Martorell N., Vilella E., Gallart-Palau X., Serra A. (2022). Industrial By-Products As a Novel Circular Source of Biocompatible Extracellular Vesicles. Adv. Funct. Mater..

[20] Graykowski D.R., Wang Y.Z., Upadhyay A., Savas J.N. (2020). The Dichotomous Role of Extracellular Vesicles in the Central Nervous System. iScience.

[21] Herrmann I.K., Wood M.J.A., Fuhrmann G. (2021). Extracellular vesicles as a next-generation drug delivery platform. Nat. Nanotechnol..

[22] Aharon A., Spector P., Ahmad R.S., Horrany N., Sabbach A., Brenner B., Aharon-Peretz J. (2020). Extracellular Vesicles of Alzheimer’s Disease Patients as a Biomarker for Disease Progression. Mol. Neurobiol..

[23] Muraoka S., DeLeo A.M., Sethi M.K., Yukawa-Takamatsu K., Yang Z., Ko J., Hogan J.D., Ruan Z., You Y., Wang Y. (2020). Proteomic and biological profiling of extracellular vesicles from Alzheimer’s disease human brain tissues. Alzheimers Dement..

[24] Gratpain V., Mwema A., Labrak Y., Muccioli G.G., van Pesch V., des Rieux A. (2021). Extracellular vesicles for the treatment of central nervous system diseases. Adv. Drug Deliv. Rev..

[25] Cristina L., María F.-R., Jose Antonio Sánchez M., María M., Julia L., Xavier G.-P., Aida S., Manash K.P. (2023). BP-EVs: A Novel Source of EVs in the Nanocarrier Field. Extracellular Vesicles—Applications and Therapeutic Potential.

[26] Wang Y., Amdanee N., Zhang X. (2022). Exosomes in schizophrenia: Pathophysiological mechanisms, biomarkers, and therapeutic targets. Eur. Psychiatry.

[27] Roy R., Lorca C., Mulet M., Sánchez Milán J.A., Baratas A., de la Casa M., Espinet C., Serra A., Gallart-Palau X. (2023). Altered ureido protein modification profiles in seminal plasma extracellular vesicles of non-normozoospermic men. Front. Endocrinol..

[28] Mun D.G., Budhraja R., Bhat F.A., Zenka R.M., Johnson K.L., Moghekar A., Pandey A. (2023). Four-dimensional proteomics analysis of human cerebrospinal fluid with trapped ion mobility spectrometry using PASEF. Proteomics.

[29] Serra A., Gallart-Palau X., Wei J., Sze S.K. (2016). Characterization of glutamine deamidation by LERLIC-MS/MS in shotgun proteomics. Anal. Chem..

[30] Gallart-Palau X., Serra A., Sze S.K. (2020). System-wide molecular dynamics of endothelial dysfunction in Gram-negative sepsis. BMC Biol..

[31] Guo X., Park J.E., Gallart-Palau X., Sze S.K. (2020). Oxidative Damage to the TCA Cycle Enzyme MDH1 Dysregulates Bioenergetic Enzymatic Activity in the Aged Murine Brain. J. Proteome Res..

[32] Sánchez Milán J.A., Fernández-Rhodes M., Guo X., Mulet M., Ngan S.C., Iyappan R., Katoueezadeh M., Sze S.K., Serra A., Gallart-Palau X. (2023). Trioxidized cysteine in the aging proteome mimics the structural dynamics and interactome of phosphorylated serine. Aging Cell.

[33] Panahi M., Hase Y., Gallart-Palau X., Mitra S., Watanabe A., Low R.C., Yamamoto Y., Sepulveda-Falla D., Hainsworth A.H., Ihara M. (2023). ER stress induced immunopathology involving complement in CADASIL: Implications for therapeutics. Acta Neuropathol. Commun..

[34] Gallart-Palau X. (2020). Systems Biology in Neuroscience: The Paramount Importance of Data Sharing and Citation. NeuroSci.

[35] Saeedi S., Israel S., Nagy C., Turecki G. (2019). The emerging role of exosomes in mental disorders. Transl. Psychiatry.

[36] Perez-Gonzalez R., Gauthier S.A., Kumar A., Levy E. (2012). The Exosome Secretory Pathway Transports Amyloid Precursor Protein Carboxyl-terminal Fragments from the Cell into the Brain Extracellular Space*. J. Biol. Chem..

[37] Levy E. (2017). Exosomes in the Diseased Brain: First Insights from In Vivo Studies. Front. Neurosci..

[38] Gallart-Palau X., Tan L.M., Serra A., Gao Y., Ho H.H., Richards A.M., Kandiah N., Chen C.P., Kalaria R.N., Sze S.K. (2019). Degenerative protein modifications in the aging vasculature and central nervous system: A problem shared is not always halved. Ageing Res. Rev..

[39] Gomes P.A., Bodo C., Nogueras-Ortiz C., Samiotaki M., Chen M., Soares-Cunha C., Silva J.M., Coimbra B., Stamatakis G., Santos L. (2023). A novel isolation method for spontaneously released extracellular vesicles from brain tissue and its implications for stress-driven brain pathology. Cell Commun. Signal.

[40] Kandimalla R., Saeed M., Tyagi N., Gupta R.C., Aqil F. (2023). Exosome-based approaches in the management of Alzheimer’s disease. Neurosci. Biobehav. Rev..

[41] Huang Y., Driedonks T.A.P., Cheng L., Rajapaksha H., Routenberg D.A., Nagaraj R., Redding J., Arab T., Powell B.H., Pletniková O. (2022). Brain Tissue-Derived Extracellular Vesicles in Alzheimer’s Disease Display Altered Key Protein Levels Including Cell Type-Specific Markers. J. Alzheimers Dis..

[42] Ruan Z., Pathak D., Venkatesan Kalavai S., Yoshii-Kitahara A., Muraoka S., Bhatt N., Takamatsu-Yukawa K., Hu J., Wang Y., Hersh S. (2020). Alzheimer’s disease brain-derived extracellular vesicles spread tau pathology in interneurons. Brain.

[43] Keerthikumar S., Chisanga D., Ariyaratne D., Al Saffar H., Anand S., Zhao K., Samuel M., Pathan M., Jois M., Chilamkurti N. (2016). ExoCarta: A Web-Based Compendium of Exosomal Cargo. J. Mol. Biol..

[44] Pathan M., Fonseka P., Chitti S.V., Kang T., Sanwlani R., Van Deun J., Hendrix A., Mathivanan S. (2018). Vesiclepedia 2019: A compendium of RNA, proteins, lipids and metabolites in extracellular vesicles. Nucleic Acids Res.

[45] Montenegro-Venegas C., Guhathakurta D., Pina-Fernandez E., Andres-Alonso M., Plattner F., Gundelfinger E.D., Fejtova A. (2022). Bassoon controls synaptic vesicle release via regulation of presynaptic phosphorylation and cAMP. EMBO Rep..

[46] Yap K., Drakew A., Smilovic D., Rietsche M., Paul M.H., Vuksic M., Del Turco D., Deller T. (2020). The actin-modulating protein synaptopodin mediates long-term survival of dendritic spines. eLife.

[47] Goldie B.J., Dun M.D., Lin M., Smith N.D., Verrills N.M., Dayas C.V., Cairns M.J. (2014). Activity-associated miRNA are packaged in Map1b-enriched exosomes released from depolarized neurons. Nucleic Acids Res..

[48] Henstridge C.M., Sideris D.I., Carroll E., Rotariu S., Salomonsson S., Tzioras M., McKenzie C.-A., Smith C., von Arnim C.A.F., Ludolph A.C. (2018). Synapse loss in the prefrontal cortex is associated with cognitive decline in amyotrophic lateral sclerosis. Acta Neuropathol..

[49] Casaletto K.B., Lindbergh C.A., VandeBunte A., Neuhaus J., Schneider J.A., Buchman A.S., Honer W.G., Bennett D.A. (2022). Microglial Correlates of Late Life Physical Activity: Relationship with Synaptic and Cognitive Aging in Older Adults. J. Neurosci..

[50] Lieberman J.A., Girgis R.R., Brucato G., Moore H., Provenzano F., Kegeles L., Javitt D., Kantrowitz J., Wall M.M., Corcoran C.M. (2018). Hippocampal dysfunction in the pathophysiology of schizophrenia: A selective review and hypothesis for early detection and intervention. Mol. Psychiatry.

[51] Abi-Dargham A., Moore H. (2003). Prefrontal DA transmission at D1 receptors and the pathology of schizophrenia. Neuroscientist.

[52] Jain R.W., Yong V.W. (2022). B cells in central nervous system disease: Diversity, locations and pathophysiology. Nat. Rev. Immunol..

[53] Tin S.K., Xu Q., Thumboo J., Lee L.Y., Tse C., Fong K.Y. (2005). Novel brain reactive autoantibodies: Prevalence in systemic lupus erythematosus and association with psychoses and seizures. J. Neuroimmunol..

[54] Dalmau J., Lancaster E., Martinez-Hernandez E., Rosenfeld M.R., Balice-Gordon R. (2011). Clinical experience and laboratory investigations in patients with anti-NMDAR encephalitis. Lancet Neurol..

[55] Glass L.J., Sinclair D., Boerrigter D., Naude K., Fung S.J., Brown D., Catts V.S., Tooney P., O’Donnell M., Lenroot R. (2017). Brain antibodies in the cortex and blood of people with schizophrenia and controls. Transl. Psychiatry.

