# Supplementary material for: Next-Generation Proteomics of Brain Extracellular Vesicles in Schizophrenia Provide New Clues on the Altered Molecular Connectome
Source: Biomedicines. 2024 Jan 8;12(1):129. doi: 10.3390/biomedicines12010129 (PMC10812948; doi:10.3390/biomedicines12010129)
Supplement: Supplementary file 1 [file biomedicines-12-00129-s001.zip › Supplementary Materials.pdf]

Summary Table:

Content	Page
**Supplementary Table S1:** Proteins exclusively identified in brain extracellular vesicles (bEVs) of subjects affected by Schizophrenia (SZ) in all analyzed brain regions.	S3
**Supplementary Table S2:** Proteins exclusively identified in brain extracellular vesicles (bEVs) of control (C) subjects in all analyzed brain regions.	S6
**Supplementary Table S3:** Proteins exclusively identified in bEVs of the PFC region in SZ.	S14
**Supplementary Table S4**. Proteins exclusively identified in bEVs of the HC in SZ.	S17
**Supplementary Table S5**. Proteins exclusively identified in bEVs of the CAU in SZ.	S25
**Supplementary Dataset S1:** List of identified proteins in bEVs of C brains in each brain region.	Additional file
**Supplementary Dataset S2:** List of identified proteins in bEVs of SZ samples in each brain region.	Additional file
**Supplementary Dataset S3:** List of identified proteins in bEVs of Control and SZ samples common to all brain regions.	Additional file
**Supplementary Dataset S4:** Data of the connectome analysis shown in Figure 4.	Additional file

Supplementary Table S1. Proteins exclusively identified in brain extracellular vesicles (bEVs) of subjects affected by Schizophrenia (SZ) in all analyzed brain regions.

	PFC		HC		CAU	
**Protein**	**Mean**	**SD**	**Mean**	**SD**	**Mean**	**SD**
TUBB8B	64,6666667	56,0119035	121	10,5830052	73,3333333	63,5400136
H7C0C1	4	4,58257569	1,33333333	1,15470054	6,33333333	4,04145188
GNG3	14,6666667	13,3166562	5,33333333	8,38649708	4,33333333	6,65832812
ATP6V1E1	7,66666667	6,65832812	5,66666667	4,04145188	2,66666667	2,51661148
CDC5L	1	1	2,66666667	0,57735027	2	1
ADGRL1	1,33333333	1,52752523	2,33333333	1,52752523	1,66666667	0,57735027
STX16-NPEPL1	2	1,73205081	5	1	2,66666667	0,57735027
STX16	2	1,73205081	5	1	2,66666667	0,57735027
SRSF3	1,33333333	1,52752523	1,33333333	1,52752523	0,66666667	0,57735027
CSNK1E	2,66666667	2,30940108	2,66666667	2,51661148	0,66666667	0,57735027
HNRNPR	3	3,60555128	3,66666667	0,57735027	3,66666667	1,52752523
ZDHHC5	2,66666667	2,51661148	3,66666667	0,57735027	1,66666667	1,52752523
EEF1B2	1	1	2	1	1,33333333	0,57735027
RPLP1	2,66666667	3,05505046	1,33333333	1,15470054	2,66666667	3,05505046
CRAT	5	7,81024968	1,33333333	1,52752523	3,33333333	2,88675135
SLC2A13	2,33333333	2,081666	2	1,73205081	0,66666667	0,57735027
CACNA1B	1	0	1,33333333	0,57735027	1,66666667	1,52752523
CD44	0,66666667	0,57735027	8	3	5	5,29150262
KBTBD11	3	2,64575131	4	1,73205081	2	1
RPS8	2	2,64575131	1,33333333	0,57735027	0,66666667	0,57735027
LMNA	13	20,7846097	0,66666667	0,57735027	1,33333333	1,52752523
PHLPP1	1,33333333	1,15470054	0,66666667	0,57735027	1	1

PTPN23	0,66666667	0,57735027	5,33333333	1,52752523	2	1
ETFA	4,66666667	5,03322296	6,33333333	2,88675135	2,33333333	1,15470054
FAM120C	2	1,73205081	2	1	1,33333333	0,57735027
JUND	3	3,60555128	1	1	2,33333333	2,081666
PRAF2	1	1	1,33333333	1,15470054	0,66666667	0,57735027
EIF4G1	2,33333333	2,081666	4	3,46410162	3,33333333	4,163332
CCT2	4	5,29150262	13	9,53939201	4	2,64575131
CTSL	4,33333333	3,7859389	2,33333333	2,51661148	2,66666667	2,51661148
CNDP2	10,3333333	11,6761866	10	3,60555128	8,33333333	3,51188458
RAB1C	3,66666667	1,15470054	4,66666667	4,163332	3	2
FARSA	5,33333333	5,03322296	4	3	2,33333333	1,52752523
PIN1	1,66666667	2,081666	4	1,73205081	1,66666667	2,081666
CAPZA1	6,66666667	1,52752523	3,33333333	0,57735027	2	2
A0A2R8YDH4	0,66666667	0,57735027	4,66666667	2,88675135	0,66666667	0,57735027
TJP2	0,66666667	0,57735027	4,66666667	2,88675135	0,66666667	0,57735027
FAM114A2	1	1	0,66666667	0,57735027	3,33333333	1,15470054
SRGAP2	1,33333333	1,15470054	3,33333333	2,30940108	0,66666667	0,57735027
DNM3	2,33333333	2,081666	5,33333333	1,52752523	1,33333333	1,15470054
FMN2	1,33333333	1,52752523	1,33333333	0,57735027	1	0
NUDCD3	3	3	2,66666667	0,57735027	2	2
SYNPR	5,33333333	4,72581563	30,6666667	7,09459888	4,33333333	5,85946528
CAMK2B	2,66666667	2,51661148	13	7,81024968	5,66666667	1,52752523
TAC3	2,33333333	2,081666	5,33333333	2,51661148	8	1
MAPRE3	4	3,60555128	7	3,46410162	5,66666667	5,13160144
MMP17	2,33333333	2,51661148	1,33333333	0,57735027	1	1
KHSRP	3,33333333	3,51188458	7,66666667	0,57735027	2	1,73205081

PDCD5	2,33333333	2,081666	5,66666667	1,52752523	2	2
HMGB1	2,33333333	3,21455025	1,66666667	1,52752523	3,33333333	3,05505046
GATD3B	0,66666667	0,57735027	4,33333333	1,52752523	1,33333333	1,15470054
DYNC1H1	1,33333333	1,52752523	7,66666667	3,51188458	1	1
MRVI1	2	2	2	1	1	1
NBEA	1	1	2,66666667	0,57735027	0,66666667	0,57735027
NLGN2	2,66666667	3,05505046	1,33333333	1,15470054	3	2
HSPA12A	1,33333333	1,15470054	3,66666667	2,51661148	1,66666667	1,52752523
KIF21A	2	2,64575131	8,33333333	2,88675135	1,33333333	1,52752523
RAD23B	6,33333333	10,1159939	10,3333333	3,7859389	1,66666667	1,15470054
DNM2	2,66666667	2,30940108	8	1	1,66666667	1,52752523
PKP4	1	1	1,33333333	1,15470054	1	1
SH3KBP1	1	1	0,66666667	0,57735027	4,66666667	0,57735027
OGDH	1,33333333	1,52752523	1	1	1,33333333	0,57735027
ARHGDIA	4,66666667	5,50757055	2,33333333	1,52752523	3	1
FLOT2	0,66666667	0,57735027	8,66666667	1,15470054	4,66666667	2,081666
MAST3	0,66666667	0,57735027	1	1	2	2,64575131
CORO1A	5,66666667	8,96288644	4,33333333	4,163332	1	1

Supplementary Table S2. Proteins exclusively identified in brain extracellular vesicles (bEVs) of control (C) subjects in all analyzed brain regions.

	PFC		HC		CAU	
Protein	Mean	SD	Mean	SD	Mean	SD
ATP1A2	12,3333333	10,7857931	3,66666667	3,21455025	11,6666667	15,30795
HSP90AB1	9	3,46410162	8,66666667	8,08290377	7,33333333	6,65832812
GAP43	19	7,21110255	10,3333333	9,60902354	26	14
GDI1	11,3333333	3,05505046	2,33333333	2,51661148	10,3333333	9,29157324
NDRG2	19,6666667	3,51188458	5	7,81024968	12,3333333	11,2398102
SEPTIN7	12	3,60555128	6,66666667	4,50924975	9,33333333	6,35085296
CAPRIN1	25,3333333	5,85946528	6,33333333	7,76745347	11,3333333	7,5055535
KRT6C	30	13,453624	34,3333333	36,6924152	29,6666667	38,370996
KRT6A	30,3333333	14,0118997	33,6666667	35,6417358	29	37,2424489
ACO2	5,33333333	0,57735027	2,33333333	2,51661148	7,33333333	2,51661148
FSCN1	7	1	2,33333333	0,57735027	5,66666667	3,51188458
VSNL1	6,66666667	4,04145188	0,66666667	0,57735027	2,66666667	1,52752523
TFG	9,66666667	3,05505046	22,3333333	11,0151411	17,3333333	3,51188458
MAP6D1	14,6666667	1,52752523	5,33333333	5,03322296	3,66666667	3,21455025
GOT2	12	1	5,66666667	3,21455025	13,6666667	4,93288286
UPF1	3,66666667	2,081666	2,33333333	1,15470054	1,66666667	0,57735027
HSPA1B	11,6666667	7,02376917	3,66666667	3,21455025	8,33333333	7,23417814
HSPA1A	11,6666667	7,02376917	3,66666667	3,21455025	8,33333333	7,23417814
LGALSL	10,6666667	7,57187779	2,66666667	2,88675135	1,33333333	0,57735027
HSPA5	7,66666667	2,081666	3,33333333	2,88675135	14,6666667	9,07377173
SLC4A10	4,66666667	2,51661148	4,33333333	4,04145188	1,33333333	1,15470054

ITSN1	4,66666667	3,21455025	6,33333333	1,15470054	5	5
ALDH2	4	2,64575131	0,66666667	0,57735027	6	6,244998
DDX6	5,33333333	1,52752523	6	3,60555128	4,66666667	5,03322296
NEFH	8	2,64575131	8,66666667	4,04145188	4,66666667	0,57735027
KRT13	26	8,54400375	10	6,244998	11	3
GDA	4	1	1,33333333	0,57735027	5,33333333	3,05505046
AGFG1	10	4,58257569	11,6666667	6,65832812	10	1
SYT1	5,33333333	2,88675135	4,33333333	5,13160144	7,33333333	3,7859389
KRT8	10	10	15	3	10,6666667	4,04145188
S100A1	2,33333333	0,57735027	2	0	2	0
PTGDS	3,66666667	1,15470054	3,33333333	1,52752523	3,33333333	2,30940108
RAB3C	6	1	3,33333333	0,57735027	3,33333333	3,51188458
LAMTOR5	3,66666667	3,05505046	3	3	3,33333333	0,57735027
GDI2	5,33333333	1,52752523	1,66666667	1,52752523	4	3,60555128
HSPA9	5	1	2,66666667	2,88675135	8,66666667	4,61880215
YTHDF3	6,66666667	1,15470054	4,33333333	1,15470054	3,66666667	1,15470054
SYNCRIP	2,66666667	1,52752523	4,33333333	1,52752523	2,66666667	3,05505046
DCLK1	5,66666667	3,51188458	8	3,60555128	2,33333333	2,51661148
SLC1A3	7,33333333	1,15470054	11	5,29150262	6,66666667	5,85946528
LGALS1	5,66666667	3,05505046	6	1	10,3333333	4,04145188
COX5B	10	4,58257569	6,33333333	6,5064071	9,33333333	3,51188458
SLC9A1	4	1,73205081	3	1	3	3,60555128
SCN2A	4,66666667	3,7859389	4	4,35889894	1,33333333	0,57735027
CRYAB	4,66666667	4,163332	2,33333333	3,21455025	3,33333333	4,163332
TPP1	4,66666667	1,15470054	2,33333333	1,15470054	2	1
ACOT7	3	2,64575131	1,33333333	1,52752523	3	3

ATP5ME	2,66666667	1,15470054	2,66666667	2,51661148	1,66666667	1,52752523
ATP6AP2	5	2,64575131	2,66666667	0,57735027	5,33333333	2,081666
DDAH1	9,66666667	3,7859389	6	5,56776436	14,3333333	5,77350269
EIF2S3	5,66666667	1,15470054	1,66666667	2,081666	2	1
EIF2S3B	5,66666667	1,15470054	1,66666667	2,081666	2	1
NDUFA5	5,33333333	2,30940108	1,33333333	1,15470054	4,33333333	4,04145188
KRT4	10,6666667	2,081666	11,6666667	4,04145188	7	2,64575131
SYAP1	5	1,73205081	5	3,60555128	3,33333333	3,21455025
UBL7	4,66666667	0,57735027	5	2,64575131	2,66666667	2,51661148
KRT73	12	5	6,66666667	6,11010093	5,66666667	2,081666
KRT74	6,33333333	6,02771377	6,66666667	6,11010093	5,66666667	2,081666
NCL	6,66666667	2,081666	4	6,08276253	1,66666667	2,081666
MRTFB	1,66666667	2,081666	6	3,46410162	8,33333333	2,081666
COX7A2	3	1	2,33333333	1,15470054	3,33333333	0,57735027
ADCY9	2,66666667	1,15470054	2,33333333	0,57735027	1,33333333	1,15470054
EIF4H	5	3	2	2	4	2,64575131
DLST	1,33333333	1,15470054	0,66666667	0,57735027	2,66666667	1,52752523
DBI	6,33333333	3,21455025	5,33333333	3,7859389	1,66666667	2,081666
PDIA3	2	1	1,33333333	1,52752523	3,33333333	3,51188458
GORASP2	7,33333333	3,21455025	10,6666667	8,326664	11,3333333	8,08290377
SKP1	3,66666667	1,52752523	2,33333333	2,51661148	4	3,60555128
SMAP1	5,33333333	1,15470054	6	5	3,66666667	2,30940108
IER3IP1	2,33333333	1,15470054	1,33333333	1,52752523	3,33333333	1,52752523
BTF3	1	1	1,33333333	0,57735027	1	1
ACTR1A	2,33333333	1,15470054	4,33333333	2,51661148	5,66666667	6,35085296
DAD1	3,33333333	0,57735027	1	1	3,33333333	1,15470054

CKMT1A	5	0	5,66666667	1,52752523	10	3
CRH	4,33333333	3,21455025	1,33333333	1,52752523	1,66666667	1,52752523
PHACTR1	1,66666667	2,081666	2	2	4,33333333	0,57735027
GSN	5,33333333	2,081666	5	5,56776436	2,33333333	2,51661148
S100B	6	0	5,66666667	1,52752523	6,33333333	2,51661148
CDH8	3	2	1,66666667	0,57735027	1	0
SH3GL2	5,66666667	4,61880215	4	3,46410162	4,66666667	3,05505046
ATP5PF	9,33333333	7,5055535	17,3333333	11,06044	15	5,56776436
GNG12	3	1	4	3,46410162	2,66666667	3,05505046
ACTN1	3	2	2,33333333	0,57735027	9,66666667	8,326664
FAM241B	8	0	7	2,64575131	10	1
PARK7	4,66666667	1,52752523	1,66666667	1,52752523	6	3
SLC17A7	4,33333333	0,57735027	9,33333333	9,01849951	2,33333333	2,51661148
AP3D1	2	1,73205081	7	4,35889894	3,66666667	2,081666
KCNQ2	1,66666667	2,081666	3,66666667	2,30940108	1	1
MTPN	2	0	2,33333333	2,081666	2,66666667	2,51661148
SNRNP70	3	0	2,33333333	1,52752523	2,33333333	0,57735027
NES	2,66666667	2,51661148	2,33333333	2,081666	2	1,73205081
WDR13	1,66666667	2,081666	1	1	0,66666667	0,57735027
STAM2	3,33333333	2,081666	1,33333333	1,52752523	1,33333333	1,52752523
SGTB	3,66666667	2,081666	4	4	1,66666667	1,52752523
NDUFS5	3,66666667	1,15470054	3	0	5,66666667	4,61880215
CHGA	5	2,64575131	1,33333333	1,15470054	4	2
NDUFA4	3	1,73205081	0,66666667	0,57735027	1,66666667	1,52752523
SYNE1	4,66666667	0,57735027	1	1	2,33333333	2,51661148
SNX2	1,33333333	1,15470054	3,66666667	1,52752523	4,33333333	2,51661148

SQSTM1	1,66666667	0,57735027	2	1	1,66666667	0,57735027
FABP3	2,66666667	0,57735027	2,66666667	2,51661148	3,66666667	2,081666
ESYT2	1,66666667	0,57735027	1	0	1,33333333	0,57735027
RBMXL1	3	2,64575131	2	1,73205081	2	2,64575131
ATP6V0D1	2,66666667	2,51661148	1	1	4	3,46410162
WIPF3	1,66666667	1,52752523	22,6666667	18,0092569	5	2
INF2	1,66666667	1,52752523	3,33333333	2,88675135	16	10,5356538
COX5A	5,33333333	6,80685929	1	1	7,66666667	4,93288286
GABRA1	2,33333333	2,081666	2	1	1	0
PTPRZ1	5,33333333	2,51661148	1,66666667	2,081666	4	4,58257569
NEDD8	0,66666667	0,57735027	1	1	2,66666667	1,52752523
BCAN	7,66666667	4,163332	18,6666667	8,50490055	12,6666667	3,05505046
LANCL1	3,66666667	1,15470054	2	1	3,33333333	1,52752523
CHMP4B	3,66666667	4,61880215	1,66666667	1,52752523	4,33333333	3,21455025
CEP170	2,66666667	1,52752523	2,33333333	1,52752523	1,66666667	2,081666
SH3BGRL	3,66666667	0,57735027	1	1	1,66666667	1,52752523
NCAN	3,33333333	2,081666	1,33333333	1,52752523	1,66666667	2,081666
DKK3	1,33333333	0,57735027	1,66666667	0,57735027	3	1
SARNP	3,66666667	1,52752523	2	2	2,66666667	2,30940108
NDUFV2	4	1	1	1	4,33333333	4,50924975
MT-CO2	1,33333333	0,57735027	2,66666667	1,52752523	2,66666667	1,52752523
APP	2,33333333	1,52752523	1,66666667	0,57735027	1,33333333	0,57735027
FABP4	1	1	1	1	1	1
PMP2	1,33333333	0,57735027	1	1	1	1
CD9	1	0	1,66666667	1,52752523	1,66666667	2,081666
CACNB1	3,66666667	1,15470054	5	2	3,66666667	1,15470054

MLC1	2,66666667	1,15470054	4,66666667	2,081666	3	0
SORBS1	4,33333333	5,77350269	6,33333333	2,88675135	8,33333333	1,52752523
TPM1	3,66666667	2,51661148	1,33333333	1,15470054	5,33333333	5,03322296
UQCRC2	1,66666667	1,15470054	2	1	1,33333333	0,57735027
THY1	3,66666667	0,57735027	1,66666667	0,57735027	3	1
FTL	1,33333333	0,57735027	1,66666667	1,52752523	2,66666667	0,57735027
CBL	3,66666667	1,15470054	3,33333333	0,57735027	4,66666667	0,57735027
PALM2AKAP2	2	1	1,33333333	1,52752523	2	2,64575131
PALM2	2	1	2,66666667	1,52752523	2	2,64575131
NAPB	2	1	2	1,73205081	4	4,58257569
PENK	1,33333333	1,15470054	22	33,808283	65,3333333	7,5055535
PSMC1	2	0	0,66666667	0,57735027	1	1
DIP2B	2,33333333	0,57735027	2,33333333	0,57735027	1,66666667	0,57735027
TMED8	1,66666667	0,57735027	1,33333333	1,52752523	2	1
TIMM8A	2	0	0,66666667	0,57735027	3	2
SORBS2	3,33333333	0,57735027	5,33333333	3,21455025	13	3,46410162
AHNAK2	2	2,64575131	19,6666667	20,0083316	1,66666667	1,52752523
WASL	2,33333333	0,57735027	3,33333333	2,081666	2	1,73205081
OPA1	1	1	1	1	1	1
TBC1D5	2,33333333	1,15470054	2,33333333	1,52752523	2,33333333	0,57735027
NLGN3	1,33333333	0,57735027	1	1	0,66666667	0,57735027
GABRB3	1,66666667	1,52752523	1,33333333	1,52752523	1	1
COX7A2L	1,33333333	0,57735027	1	0	0,66666667	0,57735027
C12orf57	3,33333333	2,081666	5	4,35889894	4,33333333	1,52752523
HSPA4	2,33333333	1,52752523	1	1	2	2
PCBP2	2,33333333	1,52752523	1	1	0,66666667	0,57735027

SEC24B	0,66666667	0,57735027	1,33333333	0,57735027	1	1
ATXN2L	1	1	4	0	2,33333333	1,52752523
SNRPC	1	1	1	0	1,33333333	1,52752523
EIF5	1	1	1	1	1,33333333	0,57735027
TIMM13	4	3	3,66666667	2,081666	2,66666667	1,15470054
NAPG	1	1	1	1	3	3
TPR	1	0	3,33333333	3,21455025	3	3,46410162
CTBP1	0,66666667	0,57735027	1	1	2,33333333	1,52752523
RAN	1,33333333	0,57735027	1	1	0,66666667	0,57735027
GPM6B	1	1	3	2,64575131	1,66666667	1,52752523
DENR	3	1	5,33333333	4,04145188	2	1
CAPZB	0,66666667	0,57735027	1,33333333	1,52752523	0,66666667	0,57735027
NEDD4L	1	0	2,66666667	2,081666	2	0
EDC4	2,33333333	2,30940108	1,33333333	1,15470054	2,66666667	3,05505046
HPCA	0,66666667	0,57735027	2	1,73205081	6	3
RBSN	2,33333333	1,52752523	1,66666667	0,57735027	1,33333333	1,52752523
PLCB1	5,66666667	5,50757055	5,66666667	4,61880215	4,66666667	2,081666
SHISA7	1,33333333	0,57735027	1,33333333	1,15470054	1,33333333	1,15470054
CAPN1	1,33333333	0,57735027	1,33333333	1,15470054	1,66666667	0,57735027
DCD	6,66666667	5,50757055	6	7,21110255	7,66666667	5,6862407
AHSA1	0,66666667	0,57735027	1,33333333	0,57735027	0,66666667	0,57735027
SRI	2	2,64575131	4	3,60555128	1,66666667	2,081666
KRT16	23,3333333	24,0277617	13,3333333	11,9303534	10,6666667	9,29157324
VCP	5	5	1,33333333	1,52752523	6,66666667	5,85946528
TOMM70	2,33333333	2,081666	2	2,64575131	5	2
LMO7	4	4,58257569	4	6,08276253	16,6666667	10,6926766

UBXN7	1,33333333	1,15470054	1,66666667	2,081666	2	2
PPP1R21	1,33333333	1,15470054	3	2,64575131	2,33333333	2,51661148
GPRC5B	1,33333333	1,52752523	2	2	3,66666667	3,51188458
CCT8	2,33333333	2,51661148	3	3	2,66666667	3,05505046
TPM3	3,66666667	3,51188458	3,33333333	2,081666	7,33333333	4,93288286
MICOS13	2,33333333	2,081666	1	1	1,66666667	2,081666
MINK1	1,66666667	1,52752523	7,66666667	4,04145188	3,66666667	1,15470054
ACOT13	0,66666667	0,57735027	1,33333333	1,15470054	1	1
CRKL	1,33333333	1,52752523	1,66666667	2,081666	1,33333333	0,57735027
DDI2	0,66666667	0,57735027	1,66666667	0,57735027	1,33333333	0,57735027
NDUFAB1	2	2	3	1,73205081	5	3
PRUNE2	1	1	0,66666667	0,57735027	2	1
DBNL	1	1	2,33333333	3,21455025	1	1
LARP1	1	1	3	4,35889894	2,66666667	2,51661148
PACS1	1,66666667	2,081666	2,66666667	2,081666	1	1
RPH3A	4,33333333	5,85946528	0,66666667	0,57735027	0,66666667	0,57735027
CACNA1E	1	1	1,33333333	1,52752523	2	1
FABP5	0,66666667	0,57735027	1,66666667	1,52752523	1	1
SCOC	1	1	4,66666667	3,21455025	0,66666667	0,57735027
SHROOM3	1,33333333	1,15470054	1,66666667	1,15470054	0,66666667	0,57735027
NUTF2	1	1	4	3,60555128	1,33333333	1,52752523
ALDH6A1	0,66666667	0,57735027	1	1	1,33333333	1,15470054
WASF3	1,33333333	1,52752523	3	1,73205081	1	1
PPP1R12A	1	1	1,33333333	0,57735027	1	0

Supplementary Table S3. Proteins exclusively identified in bEVs of the PFC region in SZ.

	PFC	
**Protein**	**Mean**	**SD**
KRT6C	84,6666667	134,704615
KRT6A	92	147,390637
ATP6V1A	2	1,73205081
CSTA	34,6666667	51,6268664
GLUL	3	2,64575131
GDI2	7,33333333	9,45163125
QDPR	1,66666667	2,081666
SCN2A	1,33333333	1,52752523
GORASP2	5,66666667	5,13160144
S100A8	59,6666667	93,9804944
ARF1	1,66666667	2,081666
ARF3	1,66666667	2,081666
HEXA	2	1
UNC5C	0,66666667	0,57735027
COX5A	2,66666667	2,30940108
NEDD8	1,33333333	1,52752523
CD9	1	1
TLN2	1,33333333	1,15470054
AP2B1	1,33333333	1,52752523
PHB	0,66666667	0,57735027
H3-2	17,6666667	26,3881286

CAPN1	6	7,81024968
DCD	4,33333333	2,30940108
HSPA1L	29	44,3057558
TPM3	7,66666667	12,4230968
CRYBG2	1,66666667	1,52752523
RPH3A	3,66666667	4,72581563
LRP1	1,33333333	1,15470054
GNAI1	2	2,64575131
SLC12A5	1	1
UBAP1	0,66666667	0,57735027
PAM	1,33333333	1,52752523
NDUFB1	1	1
PDPK1	0,66666667	0,57735027
RPS19	0,66666667	0,57735027
EIF3J	6,33333333	7,09459888
GYG1	2,66666667	2,51661148
REEP2	3,66666667	3,21455025
RPL4	1,66666667	2,081666
HOPX	3	3
DYNC1I2	1,33333333	1,15470054
DPY30	1,33333333	1,15470054
EEF1G	4	4,58257569
BCAS3	1,33333333	1,52752523
GNAI3	2	2,64575131
TCEAL5	1,66666667	1,52752523
AP1B1	1,33333333	1,52752523

VTN	3,66666667	5,50757055
CLASP1	0,66666667	0,57735027
PTRHD1	0,66666667	0,57735027
DCXR	0,66666667	0,57735027
FGB	37,3333333	62,9311793
H2AFX	18,6666667	23,4591844
MSN	10,3333333	13,7961347
TPM4	6,66666667	10,6926766
PDLIM5	1	1
ERP29	1,66666667	2,081666

Supplementary Table S4. Proteins exclusively identified in bEVs of the HC in SZ.

Protein	HC	
	Mean	SD
GAP43	8	8,54400375
DCLK1	5,66666667	2,51661148
SCOC	2,66666667	2,51661148
SRI	1,66666667	1,52752523
KCNQ2	3,33333333	0,57735027
PFN2	1,33333333	1,52752523
GPM6B	1,33333333	1,52752523
SYNPO2	13,3333333	15,2752523
SQSTM1	2,66666667	1,52752523
SYNRG	1,33333333	1,52752523
PKIA	1,66666667	2,081666
ADCY9	1,33333333	1,52752523
TSC22D2	3	3
RAB3C	2,33333333	2,51661148
PSMD4	1,66666667	1,52752523
TMEM163	4,33333333	4,50924975
MARCKSL1	5,33333333	3,7859389
SGTB	5,66666667	3,05505046
APOE	2	1
SHC3	3	3
EIF3G	1,66666667	1,52752523

CNKSR2	1,33333333	1,52752523
CEP170B	2	2,64575131
LMBRD2	1	1
WDR47	6,33333333	5,03322296
PACS1	2	0
WASF3	3	3
KIAA1217	2	1
NEDD4L	2,33333333	1,52752523
BRAF	1,66666667	0,57735027
LIMCH1	2	1
STIM1	0,66666667	0,57735027
VCPIP1	1	1
HINT1	7,33333333	6,11010093
NDUFAB1	2,33333333	2,081666
SCG3	4,66666667	5,6862407
HDAC4	4	1
SHISA6	1,66666667	1,52752523
TMEM145	0,66666667	0,57735027
PALM2AKAP2	0,66666667	0,57735027
PALM2	1,33333333	1,52752523
SLC9A3R1	7,66666667	1,15470054
SFPQ	1	1
VCP	2,33333333	2,081666
TBC1D5	0,66666667	0,57735027
RAPGEF2	1	1
OPA1	1	1

ASTN1	1,33333333	1,52752523
USP10	1,33333333	1,15470054
TPR	1,66666667	0,57735027
ATP5ME	0,66666667	0,57735027
ALDH6A1	6,33333333	3,51188458
TACC2	1,33333333	0,57735027
ERC1	1,66666667	2,081666
SHROOM2	0,66666667	0,57735027
ACTR1B	8	2
ATOX1	2,33333333	0,57735027
MINDY2	1,66666667	1,52752523
ELFN1	1,33333333	1,52752523
KCTD8	1,33333333	0,57735027
RALBP1	0,66666667	0,57735027
OSBP	2,66666667	1,15470054
GRIA1	1,66666667	2,081666
PTPRZ1	1	1
DLG2	0,66666667	0,57735027
NUP214	1,66666667	2,081666
APLP1	2	1
SHROOM3	1	1
HARS	0,66666667	0,57735027
SHISA7	5,33333333	5,50757055
SORT1	1,66666667	1,15470054
SRSF5	1,33333333	0,57735027
UFM1	1,66666667	2,081666

ASAP1	2,66666667	1,15470054
CAPZB	1	1
MACF1	3	0
NDUFV3	1	1
DIDO1	0,66666667	0,57735027
GIGYF2	1,66666667	1,52752523
LUZP2	0,66666667	0,57735027
AHSA1	0,66666667	0,57735027
A0A286YES4	1	0
PLPP3	1	0
A0A286YF41	1	0
A0A286YEZ7	1	0
AFDN	0,66666667	0,57735027
GLI3	1	1
YTHDF2	3	2,64575131
RPS3	2	2
YLPM1	0,66666667	0,57735027
AHSG	2	0
PPP1R21	2,66666667	1,52752523
EIF2S3	2,66666667	2,30940108
EIF2S3B	2,66666667	2,30940108
CCT4	4,66666667	2,51661148
HSPA4L	2,33333333	2,081666
ARHGAP23	2,33333333	0,57735027
DDAH2	4,33333333	2,081666
FECH	1,33333333	1,15470054

CCT6A	6	7,93725393
HUWE1	2,66666667	2,51661148
NYAP2	1,66666667	1,52752523
PSAT1	1	0
CNTN1	2	1
CNOT3	2	2,64575131
EHD1	2,33333333	1,52752523
RAPH1	2,33333333	2,081666
GABRA3	1,33333333	1,15470054
RPS17	2,66666667	0,57735027
PRKCA	4	3,60555128
H0YHG0	3,33333333	2,51661148
SARNP	3,33333333	2,51661148
PEX5L	2,33333333	2,081666
PHACTR2	1,33333333	0,57735027
ARHGAP21	2,66666667	0,57735027
NXF1	1	1
PIANP	2	2
OXCT1	1	1
SDHD	1,66666667	1,15470054
GLS	0,66666667	0,57735027
RASA1	5,66666667	4,93288286
ACTA1	117,666667	108,932701
CKM	4,66666667	4,50924975
STUM	2,66666667	2,51661148
TEX2	3	1,73205081

CAP1	4,33333333	2,88675135
ATP1B1	1,33333333	1,15470054
WDR1	3	3
B4DLN1	2,33333333	1,52752523
MRPL12	2,33333333	1,52752523
ZFR	1	1
ERVK3-1	0,66666667	0,57735027
PMCH	2,66666667	3,05505046
PPP1R3F	1	1
MGST3	2	2,64575131
WASH6P	1	1
RAC3	1	1
FKBP15	1,33333333	1,52752523
PCMT1	3	3
DLGAP2	2,66666667	1,15470054
DLGAP4	2,66666667	1,15470054
BORCS7-ASMT	1	1
BORCS7	1	1
COX17	1	1
RGS7BP	0,66666667	0,57735027
SNTA1	2,66666667	3,05505046
LOC114483833	2,66666667	2,51661148
H2BC1	2,66666667	2,51661148
E7EVH7	1	1
KLC1	1	1
CACNA1A	1	0

UBE4B	2	1,73205081
TGFBI	1,66666667	0,57735027
ACBD3	1,33333333	1,52752523
ADGRA1	1	1
CCT3	1,66666667	1,52752523
MICALL1	1	0
CC2D1A	0,66666667	0,57735027
OCIAD1	1,66666667	1,52752523
UFC1	2	2
PDLIM2	0,66666667	0,57735027
E5RGS7	0,66666667	0,57735027
H0YBP7	0,66666667	0,57735027
H7C1D1	0,66666667	0,57735027
PSMC5	1	1
ADSS2	1	1
F8VP03	0,66666667	0,57735027
F8W031	0,66666667	0,57735027
CPLX3	2	0
PPP6R2	2,66666667	1,15470054
UCHL3	1,33333333	1,52752523
C16orf58	1,33333333	1,15470054
UBE2N	1,33333333	1,52752523
FASN	0,66666667	0,57735027
TMEM117	0,66666667	0,57735027
SLC7A8	0,66666667	0,57735027
L1CAM	1,33333333	1,52752523

SIK3	0,66666667	0,57735027
PDE1A	3,66666667	5,50757055
GNAQ	1,33333333	1,52752523
GLRX3	1	1
PCDH7	0,66666667	0,57735027
SMARCA2	0,66666667	0,57735027
SKIV2L	1	1
A0A1B0GU92	1,66666667	2,081666
A0A1B0GU03	1,66666667	2,081666
AJM1	0,66666667	0,57735027
BRD4	0,66666667	0,57735027
MYO5A	0,66666667	0,57735027
SEC22B	0,66666667	0,57735027
EXOC3L2	0,66666667	0,57735027
UBXN6	2,66666667	3,7859389
CLSTN1	1	1
SAMD14	3,33333333	3,51188458
H3BRW5	0,66666667	0,57735027
SNX4	0,66666667	0,57735027
STX12	1,33333333	1,52752523
APOA4	1	1
EIF5A	1	1
EIF5A2	1	1
EIF5AL1	1	1
SRSF2	0,66666667	0,57735027
HNRNPM	0,66666667	0,57735027

ACE2	1,66666667	1,52752523
RAPGEF1	1	1
LMOD2	1	1

Supplementary Table S5. Proteins exclusively identified in bEVs of the CAU in SZ.

	CAU	
Protein	Mean	SD
PENK	38,6666667	15,6311655
HSPA9	3,33333333	2,51661148
GDI1	1,33333333	1,52752523
TIMM13	1	1
KRT79	5,66666667	5,50757055
CMTM4	1	1
KRT76	3,66666667	3,05505046
STX1A	1	1
PHACTR1	1,33333333	1,15470054
STRN	2,66666667	2,51661148
KRT13	5,66666667	5,13160144
ARMCX2	1	0
VDAC3	1	1
FH	1	1
HPRT1	0,66666667	0,57735027
KRT73	1	1
KRT74	1	1
ATP5F1C	1	1
TIMM8A	1	1
KAZN	2	1,73205081
GMFB	1	1

FTL	1,33333333	0,57735027
CALB1	2,33333333	1,52752523
STK39	1,33333333	1,52752523
EIF5	0,66666667	0,57735027
STRN4	1	1
NDUFS5	2	2,64575131
BAG3	0,66666667	0,57735027
POLDIP3	1,33333333	0,57735027
RPL6	1,33333333	1,15470054
PPP1R9A	2,33333333	2,081666
ZFAND2B	1,66666667	0,57735027
CHMP5	2,33333333	2,51661148
GUCY1A2	1,33333333	1,15470054
MYL10	1,66666667	1,52752523
NDUFA8	1,66666667	1,52752523
CANX	1,66666667	0,57735027
MYO1C	0,66666667	0,57735027
CKMT2	3	2
HNRNPA3	1,66666667	1,52752523
KATNB1	1	1
GPM6A	0,66666667	0,57735027
NUTF2	3	4,35889894
DLAT	0,66666667	0,57735027
PCDHGB4	0,66666667	0,57735027
PCDHGB5	0,66666667	0,57735027
SLC30A3	0,66666667	0,57735027

SEPTIN2	3	4,35889894
PGAM4	9,66666667	5,50757055
TUBAL3	3,66666667	3,51188458
PCDHGB3	0,66666667	0,57735027
PCDHGA12	0,66666667	0,57735027
PCDHGB6	0,66666667	0,57735027
PCDHGA5	0,66666667	0,57735027
PCDHGA7	0,66666667	0,57735027
PCDHGA2	0,66666667	0,57735027
PCDHGB1	0,66666667	0,57735027
PCDHGA1	0,66666667	0,57735027
PCDHGC3	0,66666667	0,57735027
PCDHGB2	0,66666667	0,57735027
PCDHGA6	0,66666667	0,57735027
PCDHGA11	0,66666667	0,57735027
PCDHGA3	0,66666667	0,57735027
PCDHGB7	0,66666667	0,57735027
PCDHGA4	0,66666667	0,57735027
PCDHGC4	0,66666667	0,57735027
PCDHGA9	0,66666667	0,57735027
PCDHGA10	0,66666667	0,57735027
PCDHGA8	0,66666667	0,57735027
CTNNA1	2,33333333	0,57735027
CTNNA2	1,33333333	1,15470054
LITAF	2,33333333	2,51661148
SFXN5	1,66666667	2,081666

CAND2	0,66666667	0,57735027
SLC32A1	0,66666667	0,57735027
MAN1A1	1	1
SNTB2	0,66666667	0,57735027
RPL7A	0,66666667	0,57735027
RPS10-NUDT3	0,66666667	0,57735027
ZNFX1	0,66666667	0,57735027
LETM1	0,66666667	0,57735027
PCNT	0,66666667	0,57735027
NOS1	0,66666667	0,57735027
GLUD2	1,66666667	1,52752523
HDGFL3	1	1
CNN1	0,66666667	0,57735027
